# Bioactive Chaetoglobosins from the Mangrove Endophytic Fungus *Penicillium chrysogenum*

**DOI:** 10.3390/md14100172

**Published:** 2016-09-27

**Authors:** Song Huang, Haiyan Chen, Wensheng Li, Xinwei Zhu, Weijia Ding, Chunyuan Li

**Affiliations:** 1 College of Materials and Energy, South China Agricultural University, Guangzhou 510642, China; singsongpine@sina.cn (S.H.); wsliscau@sina.com (W.L.); zhuxw2580@sina.com (X.Z.); 2 School of Chemistry and Chemical Engineering, Guangxi University, Guangxi Colleges and Universities Key Laboratory of Applied Chemistry Technology and Resource Development, Nanning 530004, China; czyzsu@gxu.edu.cn

**Keywords:** marine mangrove fungus, chaetoglobosin, *Penicillium chrysogenum*, cytotoxicity, antifungal activity

## Abstract

A novel chaetoglobosin named penochalasin I (**1**) with a unprecedented six-cyclic 6/5/6/5/6/13 fused ring system, and another new chaetoglobosin named penochalasin J (**2**), along with chaetoglobosins G, F, C, A, E, armochaetoglobosin I, and cytoglobosin C (**3**–**9**) were isolated from the culture of *Penicillium chrysogenum* V11. Their structures were elucidated by 1D, 2D NMR spectroscopic analysis and high resolution mass spectroscopic data. The absolute configuration of compounds **1** and **2** were determined by comparing the theoretical electronic circular dichroism (ECD) calculation with the experimental CD. Compound **1** was the first example, with a six-cyclic fused ring system formed by the connection of C-5 and C-2′ of the chaetoglobosin class. Compounds **5**–**8** remarkably inhibited the plant pathogenic fungus *R. solani* (minimum inhibitory concentrations (MICs) = 11.79–23.66 μM), and compounds **2**, **6**, and **7** greatly inhibited *C. gloeosporioides* (MICs = 23.58–47.35 μM), showing an antifungal activity higher than that of carbendazim. Compound **1** exhibited marked cytotoxicity against MDA-MB-435 and SGC-7901 cells (IC_50_ < 10 μM), and compounds **6** and **9** showed potent cytotoxicity against SGC-7901 and A549 cells (IC_50_ < 10 μM).

## 1. Introduction

Chaetoglobosins are one class belonging to the cytochalasan alkaloids, containing a 10-(indol-3-yl) group and a tricyclic core in which a macrocyclic ring is commonly fused to a perhydroisoindolone moiety [1,2,3,4]. To date, about 80 chaetoglobosins have been found from some fungi belonging to the genera *Chaetomium* [5,6,7], *Discosia* [8], *Cylindrocladium* [9], *Penicillium* [10,11], *Calonectria* [12], *Diplodia* [13], and *Phomopsis* [14], most belonging to the genus *Chaetomium*. Many of them have been reported to possess diverse bioactivities, including cytotoxicity to tumor cell lines [15,16], anti-HIV [17], antimicrobial [18], phytotoxic activity [19], and so on. Endophytes are fungi or bacteria that live in the healthy tissues of living plants without causing discernible disease to the host [20]. Among them, marine mangrove endophytic fungi have attracted significant attention for their notable ability to produce metabolites with novel structures and biological activities [21,22].

**Figure 1:**
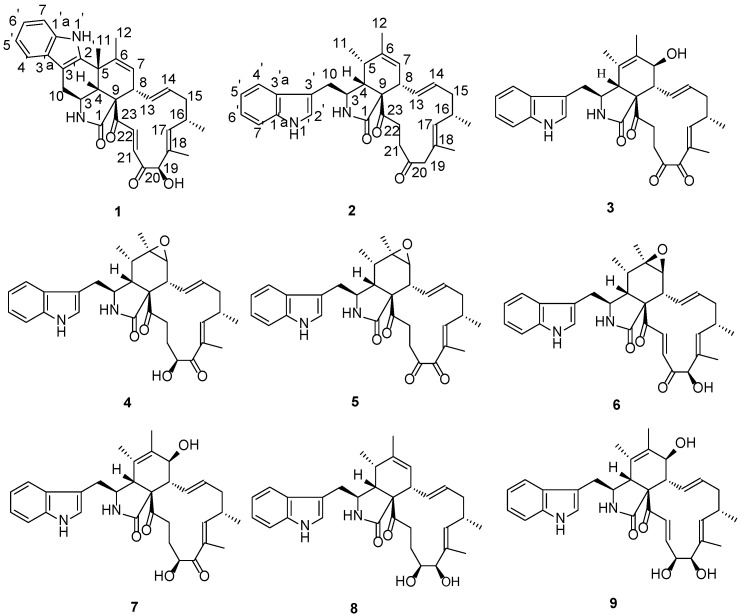
Chemical structures of the isolated compounds **1**–**9**.

*Myoporum bontioides* A. Gray, known as a semi-mangrove medicinal plant, is a small evergreen shrub distributed along the coastal regions of several Asian counties [23]. As part of our ongoing search for biologically active and/or structurally novel metabolites of the endophytic fungi from *Myoporum bontioides* [24,25], the fungus *Penicillium chrysogenum* (collection No. V11) harbored inside the normal vein attracted our interests due to its antifungal activity exhibited by the methanol extract in vitro. In this study, the chemical investigation of the fungal *Penicillium chrysogenum* V11 was carried out, and resulted in a novel chaetoglobosin termed Penochalasin I (**1**) bearing an unprecedented 6/5/6/5/6/13 six polycyclic system, and a new chaetoglobosin named Penochalasin J (**2**), along with seven known chaetoglobosins, chaetoglobosin G (**3**) [26], chaetoglobosin F (**4**) [6,27], chaetoglobosin C (**5**) [6], chaetoglobosin A (**6**) [6], chaetoglobosin E (**7**) [6], armochaetoglobosin I (**8**) [28], and cytoglobosin C (**9**) [29] (Figure 1). Details of the isolation, structure characterization, and bioactivity evaluation are reported herein.

## 2. Results and Discussion

### 2.1. Chemical Structure Elucidation

**Figure 2:**
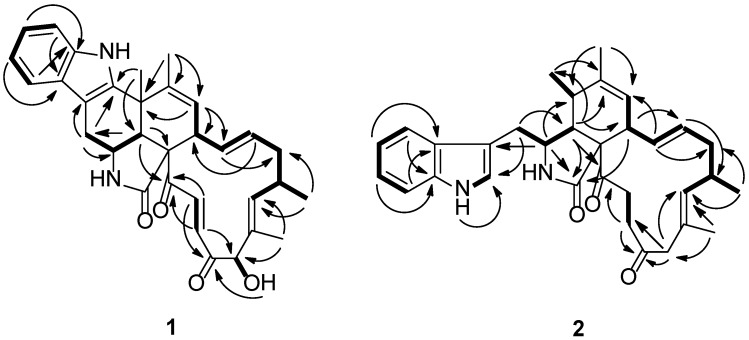
Key heteronuclear multiple bond correlation (HMBC, arrows) and chemical-shift correlation spectroscopy (COSY, bold lines) correlations of compounds **1** and **2**.

Penochalasin I (**1**) was obtained as yellow crystal. Its molecular formula was established as C_32_H_34_N_2_O_4_ by HRESIMS (high resolution electrospray ionization mass spectroscopy) at *m*/*z* 511.2614 ([M + H]^+^, cacld. for C_32_H_35_N_2_O_4_, 511.2591), indicating 17 degrees of unsaturation. The ^13^C NMR and DEPT (distortionless enhancement by polarization transfer) spectra of compound **1** (Table 1) displayed 32 carbons, including four methyls, two sp^3^ hybridized methylenes, five sp^3^ and ten sp^2^ hybridized methines, and six sp^2^ hybridized quaternary, an amide (*δ*_C_ 173.9), and two ketone (*δ*_C_ 201.7 and 198.2) carbons. The ^1^H NMR spectrum of **1** (Table 1) displayed four signals of aromatic protons at *δ*_H_ 7.44 (d, 7.8 Hz), 7.01 (td, 7.8, 1.2 Hz), 7.08 (td, 7.8, 1.2 Hz), and 7.34 (d, 8.4 Hz), along with a broad NH singlet at *δ*_H_ 10.29, suggesting the presence of a 2,3-substituted indolyl group in **1**. In addition, the ^1^H NMR spectrum showed the presence of three tertiary methyls (*δ*_H_ 1.41, 1.48, 1.66), one secondary methyl (*δ*_H_ 1.07, d, 6.6 Hz), two sets of *trans*-double bonds (H-13/14 *J* = 15.0 Hz and H-21/22 *J* = 16.8 Hz), two distinct vinyl resonances, one hydroxyl (*δ*_H_ 4.22), one oxymethine (*δ*_H_ 4.98), and an amide proton (*δ*_H_ 7.61, exchangeable). The ^13^C NMR data combined with the characteristic ^1^H NMR signals suggested that compound **1** was most likely a chaetoglobosin-based alkaloid with close resemblance to chaetoglobosin J [6]. Based on the evidence above, the main differences between compounds **1** and chaetoglobosin J were as follows: (1) The secondary 11-methyl (*δ*_H_ 1.42, d) in chaetoglobosin J had been replaced by a tertiary methyl (*δ*_H_ 1.41, s) in **1**; (2) The indolyl group in **1** had a 2,3-substituted pattern instead of the 3-substituted pattern (chaetoglobosin J). Moreover, a subsequent comparison between the ^13^C NMR spectra and degrees of unsaturation revealed by the molecular formulas of **1** and chaetoglobosin J showed that **1** had one more cyclic ring than the latter. These results suggested the connectivity of C-5 and C-2′ of **1**, which established an unprecedented 6/5/6/5/6/13 six cyclic system. This deduction was supported by HMBC (heteronuclear multiple bond correlation) correlations of H-11 to C-2′, C-4, and C-5, and of H-12 to C-5, C-6, and C-7. Finally, comprehensive HMBC and 1H-1H COSY (chemical-shift correlation spectroscopy) analysis (Figure 2) allowed the entire assignment of the proton and carbon signals for 1. To the best to our knowledge, compound 1 featured a unique carbon skeleton with a six-cyclic 6/5/6/5/6/13 fused ring system, and was the first member of the chaetoglobosin family with an unprecedented six-membered ring formed by the connection of C-5 and C-2′.

**Figure 3:**
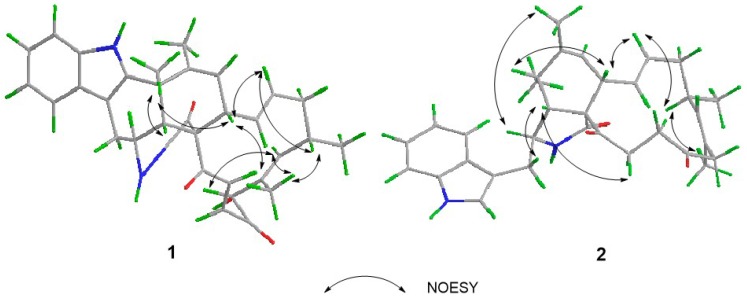
Key nuclear overhauser effect spectroscopy (NOESY) correlations of compounds **1** and **2**.

**Figure 4:**
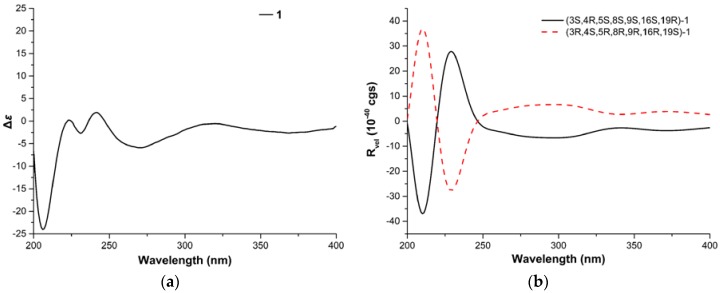
(**a**) Experimental circular dichroism (CD) of **1**, and (**b**) calculated electronic CD (ECD) of **1** in MeOH.

The relative configuration of **1** was established by analysis of its NOESY (nuclear overhauser effect spectroscopy) spectrum and proton coupling constants. NOE (nuclear overhauser effect) correlations (Figure 3) between H-8 and H-11 and between H-4 and H-11 implied that H-8 was axial and *cis* to both H-11 and H-4. The large *vicinal* coupling constant (10.2 Hz) of H-3 to H-4 implied that H-3 was *trans* to H-4. The configurations of double bonds Δ^13^ and Δ^21^ were deduced to be *E* based on their large coupling constants at 15.4 and 16.8 Hz, respectively. Consequently, NOE correlations from H-14 to H-8 and H-16 suggested H-16 was *cis* to H-8. Additionally, NOE correlations between H-16 and 18-CH_3_, and the lack of correlations between H-17 and 18-CH_3_ indicated the *E* geometry of the C-17 alkene. Therefore, NOE correlations between H-17 and H-19 indicated that H-19 was *trans* to H-16. Moreover, NOE correlations between H-8 and H-22, together with the *cis* relationship between H-4 and H-8, implied that the cyclohexene was *trans*-fused with the macrocycle moiety and *cis*-fused with the pyrrolidin-2-one. Therefore, the relative configuration of **1** was elucidated as (3S*,4R*,5S*,8S*,9S*,16S*,19R*)-**1**, shown in Figure 1. Based on the relative configuration, the absolute configuration of Penochalasin I (**1**) was determined by comparing the theoretical calculation of ECD (electronic circular dichroism) with the experimental CD. The CD of **1** (Figure 4a) is similar to the ECD of the model compound (Figure 4b), so as to determine the absolute configuration of **1** was (−)-(3*S*,4*R*,5*S*,8*S*,9*S*,16*S*,19*R*)-Penochalasin I (**1**).

Penochalasin J (**2**) was obtained as a colorless, amorphous powder. Its molecular formula (C_32_H_38_N_2_O_3_) was deduced from the positive HRESIMS data (*m*/*z* 499.2963 [M + H]^+^, calcd. 499.2955). The ^1^H NMR spectrum of **2** (Table 1) showed signals corresponding to nine olefinic and aromatic protons and four methyl groups (*δ*_H_ = 0.91, 1.23, 1.55, and 1.77 ppm), of which the five aromatic protons at *δ*_H_ 7.35 (d, 8.4 Hz), 6.99 (t, 8.4. 7.8 Hz), 7.04 (t, 8.4, 7.8 Hz), 7.56 (d, 7.8 Hz), and 7.13 (s) ppm could be assigned to a 3-substituted indolyl group. Analysis of the ^13^C NMR (Table 1) and HSQC (heteronuclear singular quantum correlation) spectroscopic data for **2** revealed 32 carbon signals, including three carbonyl groups (*δ*_C_ 210.2, 209.7.5, and 175.0), 14 olefinic and aromatic carbons, an sp^3^ quaternary carbon, five sp^3^ methylene groups, five sp^3^ methines, and four methyl groups. The protons and protonated carbon resonances in the NMR spectra of **2** were unambiguously assigned by ^1^H-^1^H COSY and HSQC experiments. The general features of its NMR data closely resembled those of penochalasin G, a cytochalasan-based alkaloid characterized from an endophytic strain of *Penicillium* sp. OUPS-19, isolated from the marine alga *Enteromorpha intestinalis* [10]. The only significant difference between the two compounds was the absence of a hydroxyl in **2** at C-19. The observed HMBC correlations (Figure 2) from H-19a and H-19b to C-17, C-18, C-18′, C-20, and C-21 confirmed the above deduction.

**Figure 5:**
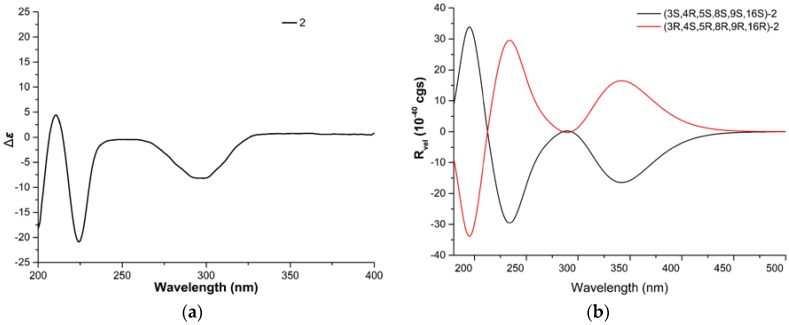
(**a**) Experimental CD of **2**, and (**b**) calculated ECD of **2** in MeOH.

The relative configuration of **2** was determined by analyzing its NOESY correlations (Figure 3) of those protons. NOE correlations between H-5 and H-8, and no observable NOE correlations between H-4 and H-8 implied that the cyclohexane ring was in a twist-boat conformation, and both H-5 and H-8 were assigned as β-orientation.^10^ Consequently, NOE correlations from H-11 to H-3 revealed their *α*-orientation. Additionally, NOE correlations between H-4 and H-10a indicated that H-3 and H-4 were not in the same orientation. Furthermore, NOE correlations between H-5 and H-8 and between H-4 and H-22b implied that the cyclohexene was *cis*-fused with the pyrrolidin-2-one and *trans*-fused with the macrocycle moiety. The *E* geometries of both the Δ^13^ and Δ^17^ double bonds in the macrocyclic ring were deduced from the large coupling constant (*J* = 15.0 Hz) and NOE correlations between H-16 and 18-CH_3_, respectively. Furthermore, NOE correlations between H-14 and H-8, together with NOE correlations between H-16 and H-14, suggested the *α*-orientation of 16-CH_3_. Therefore, the structure of **2** was elucidated as (3S*,4R*,5S*,8S*,9S*,16S*)-**2**, shown in Figure 1. The absolute configuration of Penochalasin J (**2**) was determined by comparing the theoretical calculation of ECD with the experimental CD. The CD of **2** (Figure 5a) is similar to the ECD of the model compound (Figure 5b), so as to determine the absolute configuration of **2** was (−)-(3*S*,4*R*,5S,8*S*,9*S*,16*S*)-Penochalasin J (**2**).

The structures of compounds **3**–**9** were established by comparison with the published data as chaetoglobosin G [26], chaetoglobosin F [6,27], chaetoglobosin C [6], chaetoglobosin A [6], chaetoglobosin E [6], armochaetoglobosin I [28], and cytoglobosin C [29].

More detailed spectra and ECD computational details of new compounds are available in the Supplementary Materials (Figures S1–S24 and Tables S1–S5).

### 2.2. Biological Activity

The antifungal activity of compounds **1**, **2,** and **4**–**8** were evaluated in vitro against four plant pathogens, including *Colletotrichum musae* (Berk. and M. A. Curtis) Arx. (*C. musae*), *Colletotrichum gloeosporioides* (Penz) Sacc. (*C. gloeosporioides*), *Penicillium italicum* Wehme (*P. italicm*), and *Rhizoctonia solani* Kühn (*R. solani*), and the results are summarized in Table 2. Among these, compounds **5**–**8** exhibited higher antifungal activities against *R. solani*, with MIC (minimum inhibitory concentration) values of 23.66, 11.83, 11.79, and 12.11 μM, respectively, than carbendazim (MIC 32.69 μM), which was used as the positive control. Simultaneously, compounds **2**, **6**, and **7** showed more potent activities against plant pathogen *C. gloeosporioides* with MIC values of 25.08, 47.35, and 23.58 μM, respectively, than carbendazim (MIC 65.38 μM). The results indicated that these compounds could be used as fungicides or as leads of new fungicides to the corresponding plant pathogenic fungi. Additionally, the remaining antifungal results of compounds **2** and **4**–**8** were moderate, with MIC values in the range of 48.55–100.34 μM. In comparision to other chaetoglobosins, compound **1** only showed weak antifungal activity to *R. solani* (MIC 195.98 μM), and was inactive towards another three test fungi (MIC > 391.96 μM). So, the connection between C-5 and C-2′ appears to greatly decrease the antifungal potency. In previous reports, Chaetoglobosin A (**6**) and C (**5**) showed marked inhibitory effects on the fungus *Mucor miehei* [2]. Chaetoglobosin A (**6**) also displayed significant growth inhibitory activity against the fungi *Setosphaeria turcica* [18], *Botrytis cinerea*, *Sclerotinia sclerotiorum* [30], *Rhizopus stolonifer*, and *Coniothyrium diplodiella* [31]. Chaetoglobosin V and chaetoglobosin G have been reported to exhibit high antifungal activity against *Alternaria solani* [26].

To our best knowledge, this is the first report of antifungal activities against the test plant pathogenic fungi of the seven isolated chaetoglobosins.

Compounds **1**–**9** were examined for their cytotoxic activities against three human tumor cell lines, including a human breast cancer cell line (MDA-MB-435), a human gastric cancer cell line (SGC-7901), and a human lung adenocarcinoma epithelial cell line (A549) by MTT (3-(4,5-dimethylthiazol-2-yl)-2,5-diphenyl-2*H*-tetrazolium bromide) assay, as described in the liturature [24] using epirubicin as positive control. The results are presented in Table 3. As shown in Table 3, Penochalasin I (**1**) exhibited marked cytotoxic activities against MDA-MB-435 and SGC-7901 cell lines (IC_50_ < 10 μM). Two other compounds, chaetoglobosin A (**6**) and cytoglobosin C (**9**) showed potent cytotoxicity against both SGC-7901 and A549 cell lines (IC_50_ (half maximal inhibitory concentration) < 10 μM). In contrast, armochaetoglobin I (**8**) showed no inhibitory activities against any of the test cell lines (IC_50_ > 40 μM), and chaetoglobosin E (**7**) was inactive towards both MDA-MB-435 and SGC-7901 cell lines (IC_50_ > 40 μM). The remaining compounds exhibited moderate cytotoxicity, with IC_50_ values ranging from 12.58 to 38.77 μM. Our results were consistent with the structure–activity relationships reported in previous studies [19]. Compound **5** exhibited higher cytotoxic activities against the test cell lines than compound **3**, suggesting that the epoxide ring at C-6/C-7 favoured an improvement of cytotoxicity. Compound **4** showed lower cytotoxic activities than compound **5**, indicating that the reduction of 20-carbonyl into 20-hydroxyl could decrease the cytotoxicity. This deduction could also be concluded by comparison of the cytotoxic activities between compounds **3** and **7**. In previous investigations, the known chaetoglobosins covered in the study had shown cytotoxicity against many different human cancer cell lines [15,16,27,28,29,32,33]. To our best knowledge, there were no previous reports on the cytotoxicity of these chaetoglobosins to MDA-MB-435, A549 and SGC-7901 cell lines, except compounds **8** and **9**, which were tested on the A549 cell line [28,29] with IC_50_ values almost identical to our current results.

## 3. Materials and Methods

### 3.1. General Experimental Procedures

NMR experiments were carried out on a Bruker AVIII 600 MHz NMR spectrometer (Bruker BioSpin GmbH company, Rheinstetten, Germany) (^1^H 600 MHz, ^13^C 150 MHz), with tetramethylsilane as the internal standard. Optical rotations were recorded with an MCP 300 (Anton Paar, Shanghai, China) polarimeter at 28 °C. UV spectra were measured on a PERSEE TU-1900 spectrophotometer. IR spectra were carried out on a Nicolet Nexus 670 spectrophotometer, in KBr discs. CD spectra were measured on a Chirascan™ CD spectrometer (Applied Photophysics, London, UK). ESIMS spectra were recorded on a Finnigan LCQ-DECA mass spectrometer, and HRESIMS spectra were recorded on a Thermo Fisher Scientific Q-TOF mass spectrometer. Column chromatography (CC) was performed on silica gel (200–300 mesh, Qingdao Marine Chemical Factory, Qingdao, China) and Sephadex LH-20 (Amersham Pharmacia Biotech., Uppsala, Sweden). Thin-layer chromatography (TLC) was performed on silica gel plates (Qingdao Huang Hai Chemical Group Co., Qingdao, China, G60, F-254). The high-performance liquid chromatography (HPLC) separation was performed on a Varian Prostar 210 system equipped with a Prostar 320 UV detector on a preparative Hypersil C-18 BDS column (250 × 21.2 mm, L × ID, 5 μm Varian Dynamax, Thermo Fisher Scientific Inc., Waltham, MA, USA). All other chemicals used were analytical grade.

### 3.2. Fungal Material

The strain of *Penicillium* chrysogenum V11 was isolated from the vein of *Myoporum bontioides* A. Gray in Leizhou Peninsula and deposited in the College of Materials and Energy, South China Agriculture University, Guangdong Province, China. The fungus was identified using a molecular biological protocol by DNA amplification and sequencing of the ITS region. A BLAST search result showed that the sequence was the most similar (99%) to the sequence of *Penicillium chrysogenum* (compared to KR011761.1, KX349473.1, JQ015265.1, and KF039676.1). The sequence data obtained from the fungal strain was submitted to GenBank with accession number KX777253.

### 3.3. Fermentation, Extraction, and Isolation

The fungus was fermented with a rice medium (100 mL water, 100 g rice, 0.3 g crude sea salt) in Erlenmeyer flasks (1 L × 100) at 28 °C for 30 days under static conditions.

The fungal products were extracted with methanol three times. The solvent was filtered with cheesecloth and concentrated to 2 L, then extracted with ethyl acetate (1:1, *v*/*v*) to yield 30.8 g crude extract. The extract was subjected to silica gel column chromatography and eluted with a gradient system of petroleum ether/ethyl acetate (75:25, 50:50, 25:75, 0:100, *v*/*v*) to give fractions A–D. Fraction A was chromatographed on a silica gel column, eluted with a gradient system of petroleum ether/ethyl acetate (85:15, 75:15, 50:50, *v*/*v*) to provide fractions A1, A2, and A3. Fractions A2 and A3 were subjected to Sephadex LH-20 column using dichloromethane/methanol (40:60, *v*/*v*) as eluent to give compound **2** (3 mg), and compound **3** (5 mg), respectively. Fraction B was applied to silica gel column chromatograph, with petroleum ether/ethyl acetate (75:25, 50:50, 25:75, *v*/*v*) to provide fractions B1, B2, and B3. Fraction B2 was purified by repeated CC on Sephadex LH-20 column using methanol as eluent to obtain HB1–HB3. Repeated recrystallization of fractions HB1 and HB2 at room temperature from acetone yielded compounds **4** (3 mg) and **5** (100 mg), respectively. Fraction HB3 was further separated by preparative HPLC on a C18 column (250 × 21.2 mm, L × ID, 5 μm, Varian Dynamax) eluted with a gradient system of methanol/water from 30:70 to 100:0 at a flow rate of 6 mL/min in 40 min to afford compound **1** (5 mg) at the retention time of 31.5 min. Fractions C and D were subjected to silica gel column chromatography and eluted with a gradient system of petroleum ether/ethyl acetate (25:75, 15:85, 0:100, *v*/*v*), respectively, to provide fractions C1–C3 and fractions D1–D3, respectively. Fractions C1 and D2 were purified on Sephadex LH-20 column using methanol as eluent to get fractions HC1–HC3 and fractions HD1–HD3, respectively. Repeated recrystallization of fractions HC1 and HC2 at room temperature from acetone yielded compounds **6** (8 mg) and **7** (120 mg), respectively. Fractions HD2 and HD3 were further purified by preparative HPLC on a C18 column (250 × 21.2 mm, L × ID, 5 μm, Varian Dynamax) eluted with a gradient system of methanol/water from 30:70 to 100:0 at a flow rate of 6 mL/min in 40 min to afford compounds **8** (10 mg) and **9** (4.5 mg) at the retention times of 26.2 min and 23.8 min, respectively.

Compound (**1**). Yellow crystal; ${\left[\alpha \right]}_{D}^{25}$ −64.56 (*c* 0.005, MeOH); UV (MeOH) λ_max_ (log ε) = 224 (3.26), 281 (1.57) nm; IR (KBr) ν_max_ = 3493, 3132, 3013, 1685,1615, 1386, 1078, 976, 752 cm^‒1^ ; ^1^H and ^13^C NMR data see Table 1; HRESIMS *m*/*z* 511.2614 ([M + H]^+^, cacld. for C_32_H_35_N_2_O_4_, 511.2591).

Compound (**2**). Colorless, amorphous powder. ${\left[\alpha \right]}_{D}^{25}$ −191.2 (*c* 0.005, MeOH); UV (MeOH) λ_max_ (log ε) = 220 (3.40), 283 (2.56) nm; IR (KBr) ν_max_ = 3424, 3187, 2919, 2851, 1706, 1685, 1618, 1494, 1108, 977, 740 cm^−1^; ^1^H and ^13^C NMR data see Table 1; HRESIMS *m*/*z* 499.2963 ([M + H]^+^, calcd. for C_32_H_38_N_2_O_3_ 499.2955).

### 3.4. Computational Details

The ECD spectra of Penochalasins I (**1**) and J (**2**) were calculated based on the following procedures. Because the CD of a pair of enantiomers is a mirror symmetry, and the relative configuration of **1** and **2** were determined by NOESY, the two model compounds (3*S*,4*R*,5*S*,8*S*,9*S*,16*S*,19*R*)-**1** and (3*S*,4*R*,5*S*,8*S*,9*S*,16*S*)-**2** were used in the absolute configuration determination. Systematic conformational search of the two compounds was carried out by the Vega ZZ program [34] with MMFF94 force field within a window of 7 kcal·mol^−1^. Two conformers (**1a**, 0.0 kcal·mol^−1^; **1b**, 3.76 kcal·mol^−1^) were found for (3*S*,4*R*,5*S*,8*S*,9*S*,16*S*,19*R*)-**1**, and four conformers (**2a**, 0.62 kcal·mol^−1^; **2b**, 1.12 kcal·mol^−1^; **2c**, 1.22 kcal·mol^−1^; **2d**, 0.0 kcal·mol^−1^) were found for (3*S*,4*R*,5*S*,8*S*,9*S*,16*S*)-**2**. All of the low energy conformers were further optimized by the B3LYP/6-311G (d, p). The same level harmonic vibrational frequencies were calculated to confirm their stability and to estimate their relative Gibbs free energies at 298.15 K. The ECDs of those compounds were calculated using the time-dependent density functional theory (TD-DFT) method at the PCM/PBE0/6-311++G (d, p) level. The number of singlet excited states per conformer was 40. Solvent effects were considered by the Integral Equation Formalism Polarizable Continuum Model (IEFPCM) in methanol. Finally, the ECDs of the conformers were Boltzmann weighted according to the calculated Gibbs free energies. The calculated ECD curves were generated using SpecDis1.64 (University of Würzburg, Würzburg, Germany) with σ = 0.35 ev and UV shift 10 nm. All DFT calculations were performed using the Gaussian09 program (Gaussian, Wallingford, CT, USA). All calculations were performed with the High-Performance Computing Platform of Guangxi University.

### 3.5. Antifungal Activity Assay

The following four phytopathogenic fungi were used for bioassay: *Colletotrichum musae* (Berk. and M. A. Curtis) Arx. (*C. musae*), *Colletotrichum gloeosporioides* (Penz) Sacc. (*C. gloeosporioides*), *Penicillium italicum* Wehme (*P. italicm*), *Rhizoctonia solani* Kühn (*R. solani*). They were obtained from the College of Agriculture, South China Agricultural University. The antimicrobial activities of the pure compounds against the four phytopathogenic fungi were determined by the broth dilution method as described in the previous literature to get the minimum inhibitory concentration (MIC) [26]. Carbendazim and the solvent were used as positive and negative control, respectively.

### 3.6. Cytotoxicity Assay

Cytotoxicity activities of the compounds against three human tumor cell lines, including a human breast cancer cell line (MDA-MB-435), a human gastric cancer cell line (SGC-7901), and a human lung adenocarcinoma epithelial cell line (A549) were tested by MTT method, as described in a previous report [25]. Epirubicin was used as the positive control.

## 4. Conclusions

This study led to the isolation of two new chaetoglobosins—penochalasins I (**1**) and J (**2**)—which enriched the library of natural compounds. Notably, penochalasin I (**1**) possessed a unique six-cyclic 6/5/6/5/6/13 fused ring structure and was the first example with such a system formed by the connection of C-5 and C-2′ of the chaetoglobosin class. Compounds **5**–**8** exhibited potent antifungal activities against the plant pathogen *R. solani* (MICs = 23.66, 11.83, 11.79, and 12.11 μM), and compounds **2**, **6**, and **7** displayed potent antifungal activities towards *C. gloeosporioides* (MICs = 25.08, 47.35, and 23.58 μM), with results that were better than the positive control, carbendazim. Compound **1** exhibited marked cytotoxic activities against MDA-MB-435 and SGC-7901 cell lines (IC_50_ < 10 μM), while compounds **6** and **9** showed potent cytotoxicity against both SGC-7901 and A549 cell lines (IC_50_ < 10 μM). These bioactive penochalasins in the endophytic fungus might play a defensive role by inhibiting other invasive fungi, providing more nutrition and living space. They could be worthy of consideration for the development and research of antifungal and/or antitumor agents.

## Figures and Tables

**Table 1:** ^1^H and ^13^C NMR data for compounds **1**–**2**.

No.	1 ^a^		2 ^a^	
	*δ*_C_	*δ*_H_, Mult. (*J* in Hz)	*δ*_C_	*δ*_H_, Mult. (*J* in Hz)
1	173.9, C		175.0, C	
2-NH		7.61, s		unobservable
3	53.4, CH	3.75, m (10.2)	54.6, CH	3.47, m
4	52.5, CH	3.25, d (10.2)	51.6, CH	2.62, d (3.6)
5	41.8, C		36.1, CH	2.40, m
6	142.3, C		140.4, C	
7	124.9, CH	5.19, t (1.8)	126.8, CH	5.32, m
8	41.5, CH	3.07, dt (9.6, 2.4)	48.5, CH	2.77, d (10.2)
9	61.9, C		67.5, C	
10	29.1, CH_2_	a2.55, dd (13.8, 10.8)	33.4, CH_2_	a2.80, dd (14.4, 4.8)
		b3.19, dd (13.8, 10.8)		b2.99, d (4.2)
11	23.2, CH_3_	1.41, s	14.0, CH_3_	1.23, d (7.2)
12	21.5, CH_3_	1.66, s	20.2, CH_3_	1.77, s
13	133.1, CH	5.68, qd (15.4, 10.2, 2.4)	131.8, CH	6.01, ddd (15.0, 10.2, 1.8)
14	133.7, CH	5.42, td (15.4, 10.8, 2.4)	131.8, CH	5.02, ddd (15.0, 10.8, 3.6)
15	41.1, CH_2_	a2.06, m	42.5, CH_2_	a1.84, m
		b2.45, m		b2.17, m
16	33.6, CH	2.77, m	33.2, CH	2.44, m
16-CH_3_	21.0, CH_3_	1.07, d (6.6)	21.8, CH_3_	0.91, d (6.6)
17	140.3, CH	5.60, dd (10.8, 1.8)	137.7, CH	5.07, d (9.0)
18	133.4, C		129.1, C	
18-CH_3_	11.2, CH_3_	1.48, s	16.4, CH_3_	1.55, s
19	82.7, CH	4.98, d (4.8)	53.9, CH_2_	a2.66, d (15.6)
				b3.01, d (15.6)
19-OH		4.22, d (4.8)		
20	201.7, C		209.7, C	
21	133.3, CH	6.76, d (16.8)	36.9, CH_2_	a1.80, m
				b2.21, m
22	138.8, CH	8.23, d (16.8)	37.9, CH_2_	a1.60, m
				b3.08, m
23	198.2, C		210.2, C	
1′-NH		10.29, s		10.16, s
1′a	137.8, C		137.5, C	
2′	139.9, C		125.6, CH	7.13, s
3′	108.4, C		110.5, C	
3′a	127.9, C		128.9, C	
4′	118.7, CH	7.44, d (7.8)	119.5, CH	7.56 d (7.8)
5′	119.9, CH	7.01, t (8.4, 7.8)	119.9, CH	6.99 t (8.4, 7.8)
6′	122.1, CH	7.08, t (8.4, 7.8)	122.1, CH	7.04 t (8.4, 7.8)
7′	111.9, CH	7.34, d (8.4)	112.4, CH	7.35 d (8.4)

^a^ Measured in CD_3_COCD_3_ at 600 MHz (^1^H) and 150 MHz (^13^C).

**Table 2:** The antifungal activity of the isolated compounds by the minimum inhibitory concentration (MIC) values (μM).

Compounds	*C. musae*	*P. italicum*	*R. solani*	*C. gloeosporioides*
**1**	>391.96	>391.96	195.98	>391.96
**2**	100.34	100.34	50.17	25.08
**4**	47.14	47.14	ND	94.29
**5**	94.65	94.65	23.66	94.65
**6**	94.70	94.70	11.83	47.35
**7**	94.34	23.58	11.79	23.58
**8**	96.90	48.45	12.11	ND
Carbendazim ^a^	32.69	16.34	32.69	65.38

^a^ presented as positive control; ND—not determined.

**Table 3:** Cytotoxicity (IC_50_, μM) ^a^ of compounds **1**–**9** against MDA-MB-435, SGC-7901, and A549 cell lines.

Compounds	Cell Lines		
	MDA-MB-435	SGC-7901	A549
**1**	7.55 ± 0.71	7.32 ± 0.68	16.13 ± 0.82
**2**	36.68 ± 0.90	37.70 ± 1.30	35.93 ± 0.66
**3**	38.77 ± 0.65	25.86 ± 0.84	27.63 ± 0.45
**4**	37.77 ± 0.41	26.53 ± 0.56	27.72 ± 0.81
**5**	19.97 ± 1.03	15.36 ± 0.89	17.82 ± 0.85
**6**	37.56 ± 0.95	7.48 ± 1.01	6.56 ± 0.67
**7**	>40	>40	36.63 ± 0.45
**8**	>40	>40	>40
**9**	12.58 ± 0.90	8.15 ± 0.64	3.35 ± 0.47
Epirubicin ^b^	0.56 ± 0.06	0.37 ± 0.11	0.61 ± 0.05

^a^ IC_50_ values are taken as means ± standard deviation from three independent experiments; ^b^ Used as a positive control.

## Supplementary Materials

The following are available online at www.mdpi.com/1660-3397/14/10/172/s1, 1D and 2D NMR, HRESIMS, IR, UV and ECD computational data of compounds l and 2.
