# Supplementary material for: Bioactive Chaetoglobosins from the Mangrove Endophytic Fungus Penicillium chrysogenum
Source: Mar Drugs. 2016 Sep 27;14(10):172. doi: 10.3390/md14100172 (PMC5082320; doi:10.3390/md14100172)
Supplement: Supplementary file 1 [file marinedrugs-14-00172-s001.pdf]

Supplementary Materials: Bioactive Chaetoglobosins from the Mangrove Endophytic Fungus *Penicillium chrysogenum*

Song Huang, Haiyan Chen, Wensheng Li, Xinwei Zhu, Weijia Ding and Chunyuan Li

Contents

Figure S1. ^{1}H NMR spectrum (600 MHz) of compound **1** in CD_3COCD_3 .... S2

Figure S2. ^{13}C NMR spectrum (150 MHz) of compound **1** in CD_3COCD_3 .... S2

Figure S3. DEPT90 spectrum (150 MHz) of compound **1** in CD_3COCD_3 .... S3

Figure S4. DEPT135 spectrum (150 MHz) of compound **1** in CD_3COCD_3 .... S3

Figure S5. HSQC spectrum (600/150 MHz) of compound **1** in CD_3COCD_3 .... S4

Figure S6. HMBC spectrum (600/150 MHz) of compound **1** in CD_3COCD_3 .... S4

Figure S7. Expansion of HMBC spectrum (600/150 MHz) of compound **1** in CD_3COCD_3 .... S5

Figure S8. ^{1}H-^{1}HCOSY spectrum (600 MHz) of compound **1** in CD_3COCD_3 .... S5

Figure S9. NOESY spectrum (600 MHz) of compound **1** in CD_3COCD_3 .... S6

Figure S10. HRESIMS spectrum of compound **1** S6

Figure S11. IR spectrum of compound **1** S7

Figure S12. UV spectrum of compound **1** S7

Figure S13. ^{1}H NMR spectrum (600 MHz) of compound **2** in CD_3COCD_3 .... S8

Figure S14. ^{13}C NMR spectrum (150 MHz) of compound **2** in CD_3COCD_3 .... S8

Figure S15. HSQC spectrum (600/150 MHz) of compound **2** in CD_3COCD_3 .... S9

Figure S16. HMBC spectrum (600/150 MHz) of compound **2** in CD_3COCD_3 .... S9

Figure S17. Expansion of HMBC spectrum (600/150 MHz) of compound **2** in CD_3COCD_3 .... S100

Figure S18. ^{1}H-^{1}HCOSY spectrum (600 MHz) of compound **2** in CD_3COCD_3 .... S100

Figure S19. NOESY spectrum (600 MHz) of compound **2** in CD_3COCD_3 .... S111

Figure S20. HRESIMS spectrum of compound **2** S111

Figure S21. IR spectrum of compound **2** S122

Figure S22. UV spectrum of compound **2** S122

ECD computational details of Compounds 1 and 2 .... S13

Figure S23. Cartesian coordinate of optimized (3S,4R,5S,8S,9S,16S,19R)-**1** S133

Table S1. Standard orientation of **1a** S133

Table S2. Standard orientation of **1b** S155

Figure S24. Cartesian coordinate of optimized (3S,4R,5S,8S,9S,16S)-**2** S166

Table S3. Standard orientation of **2a** S177

Table S4. Standard orientation of **2b** S18

Table S5. Standard orientation of **2c** S20

Table S6. Standard orientation of **2d** S211

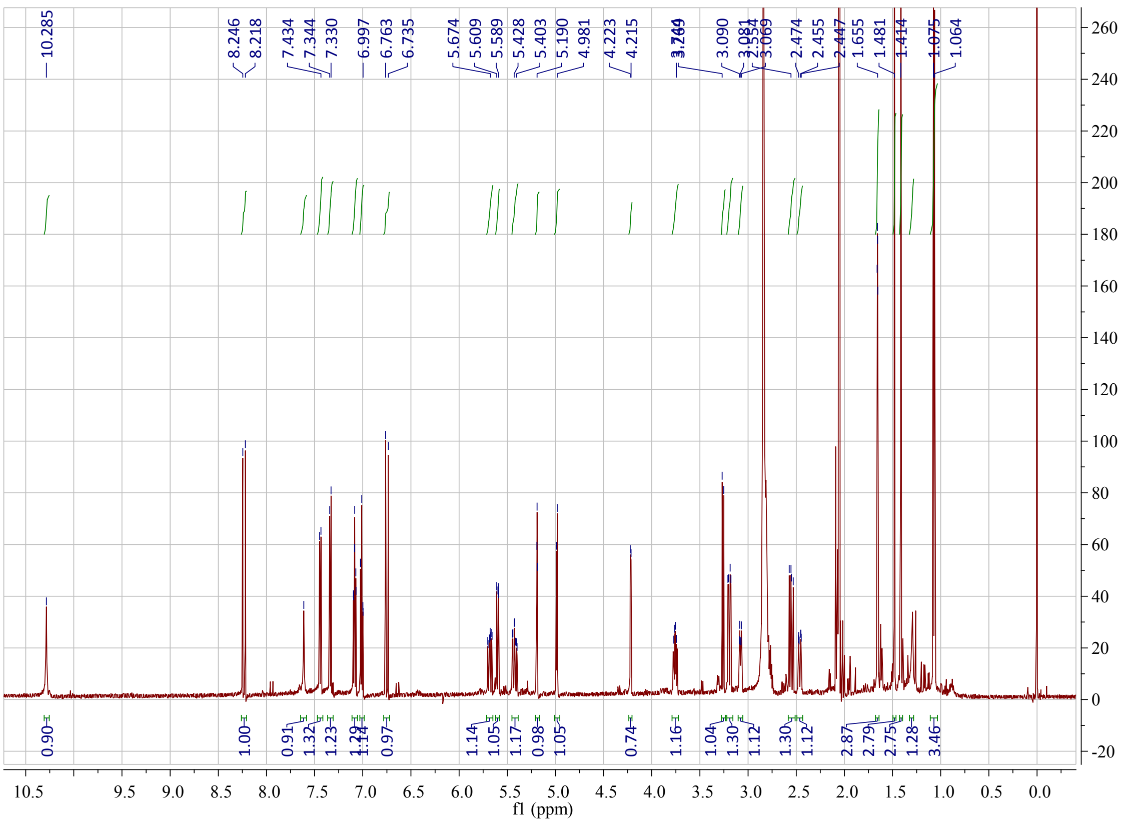

Figure S1. ^{1}H NMR spectrum (600 MHz) of compound **1** in CD_3COCD_3.

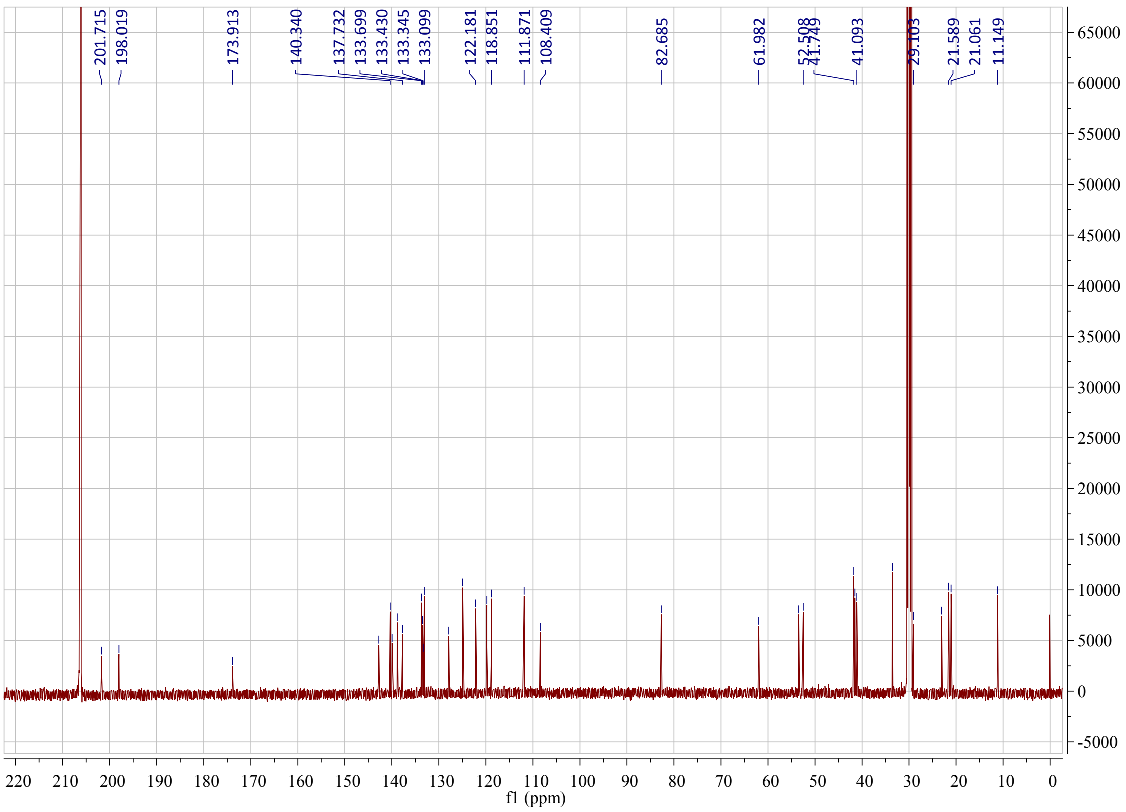

Figure S2. ^{13}C NMR spectrum (150 MHz) of compound **1** in CD_3COCD_3.

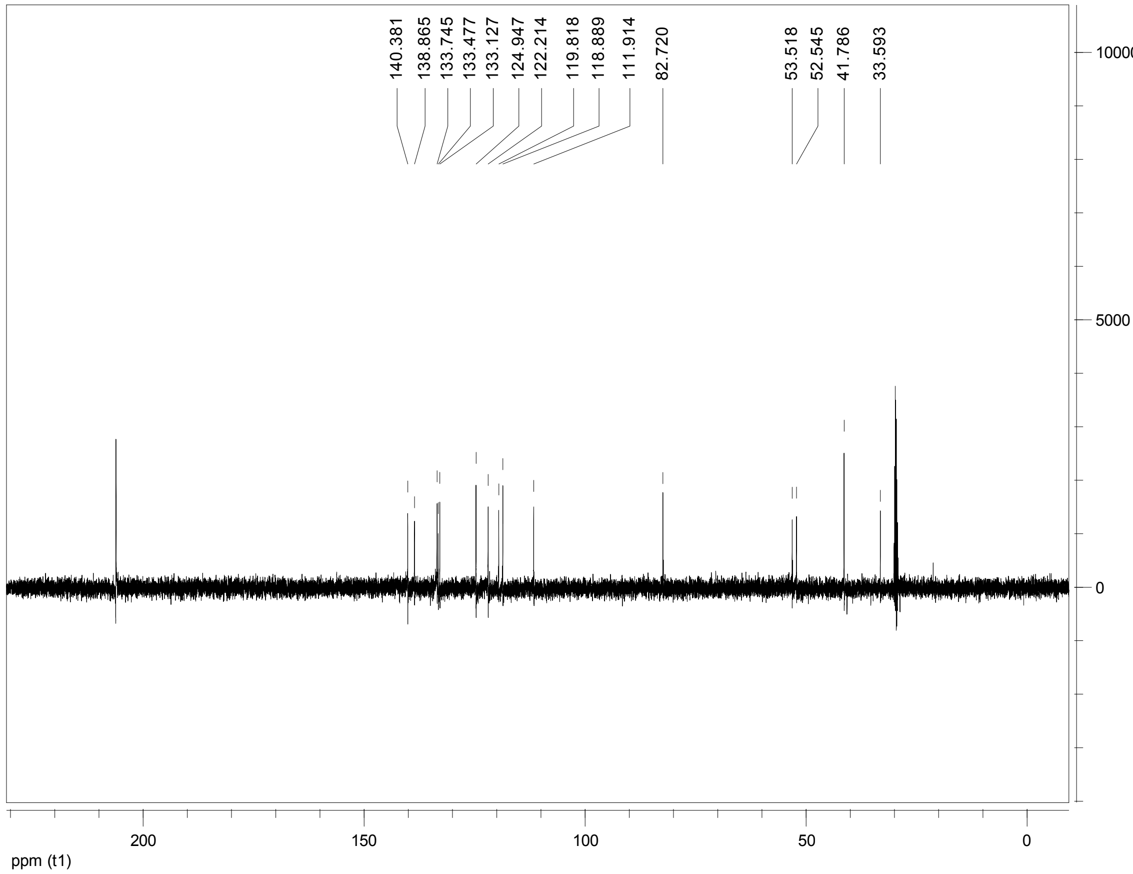

Figure S3. DEPT90 spectrum (150 MHz) of compound **1** in CD_3COCD_3.

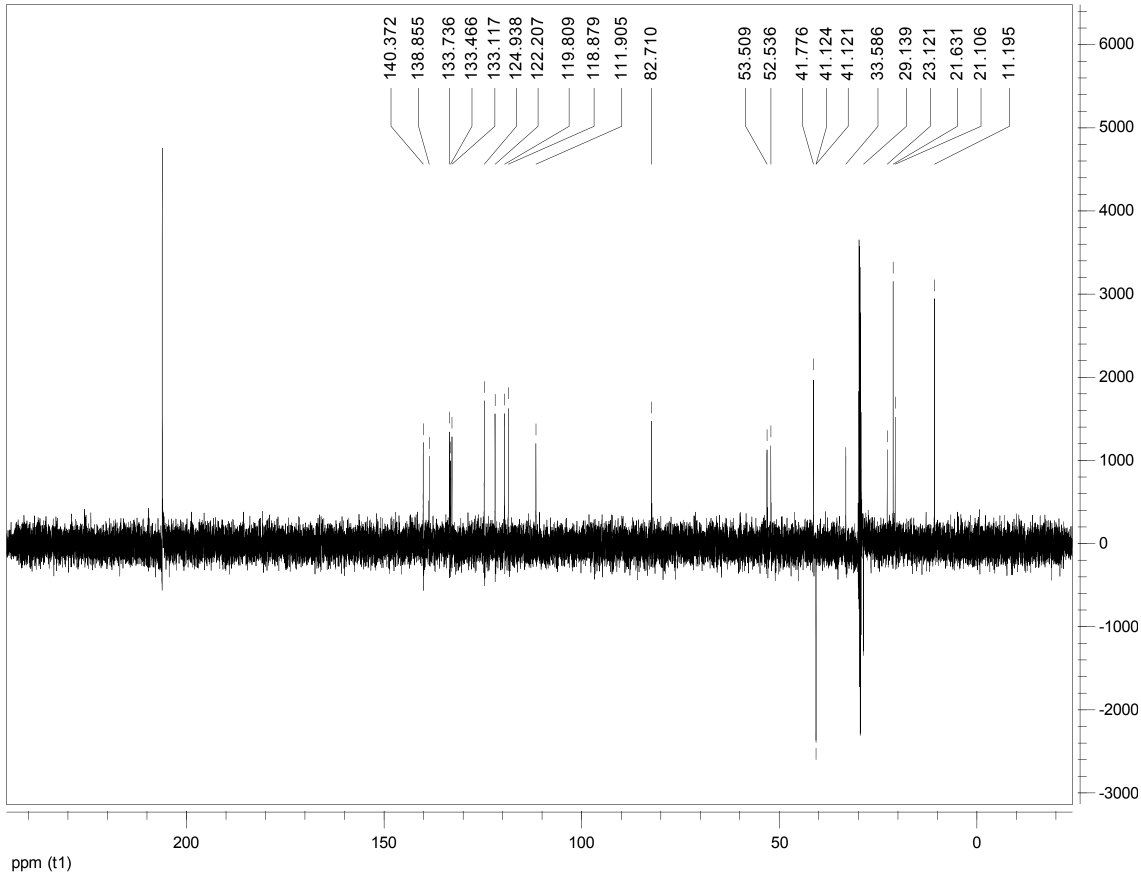

Figure S4. DEPT135 spectrum (150 MHz) of compound **1** in CD_3COCD_3.

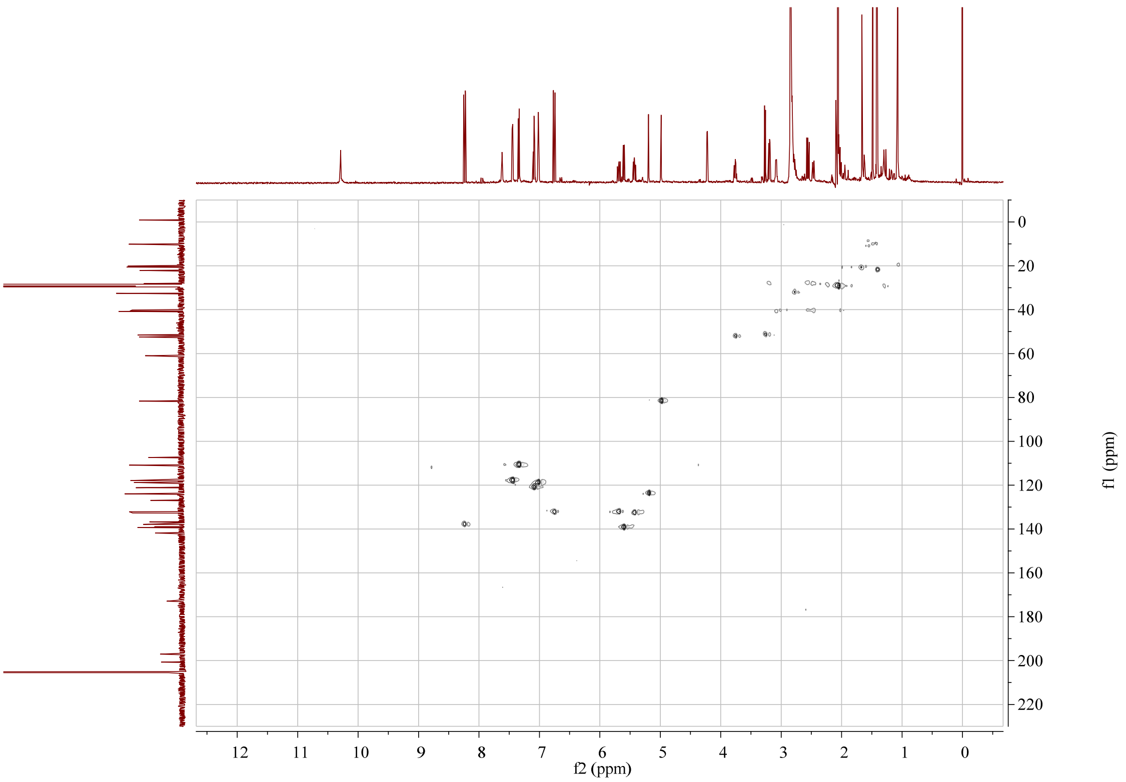

Figure S5. HSQC spectrum (600/150 MHz) of compound **1** in CD_3COCD_3.

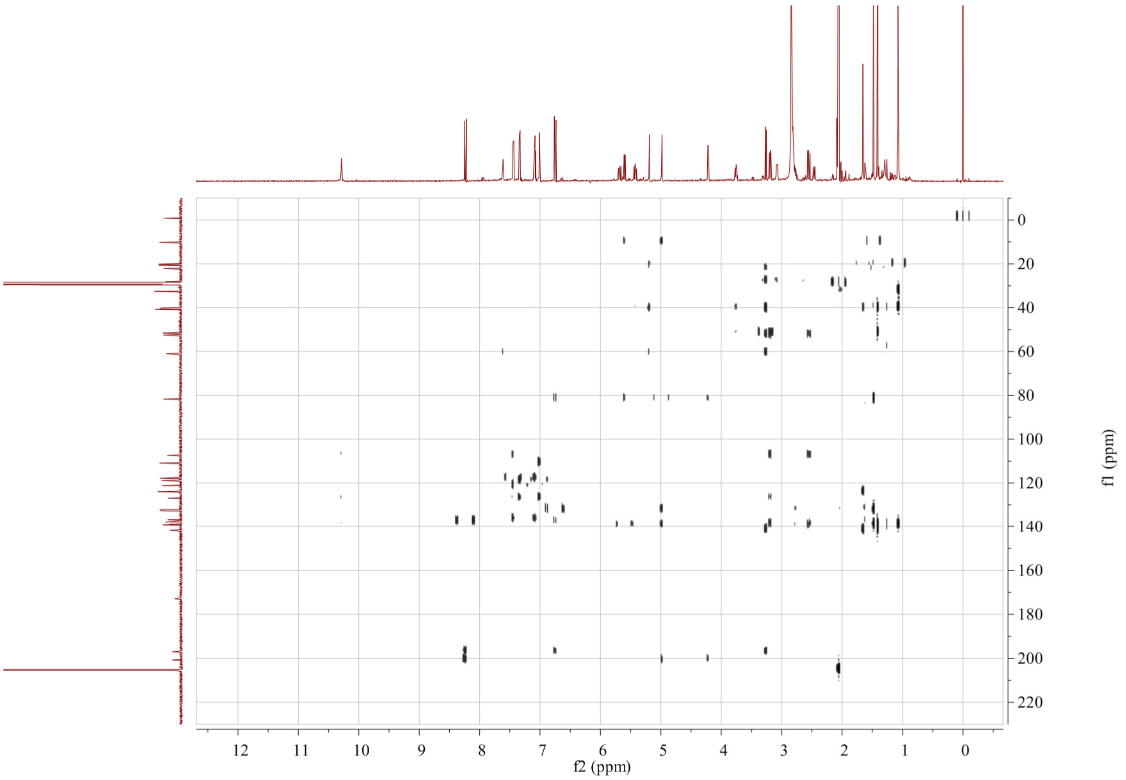

Figure S6. HMBC spectrum (600/150 MHz) of compound **1** in CD_3COCD_3.

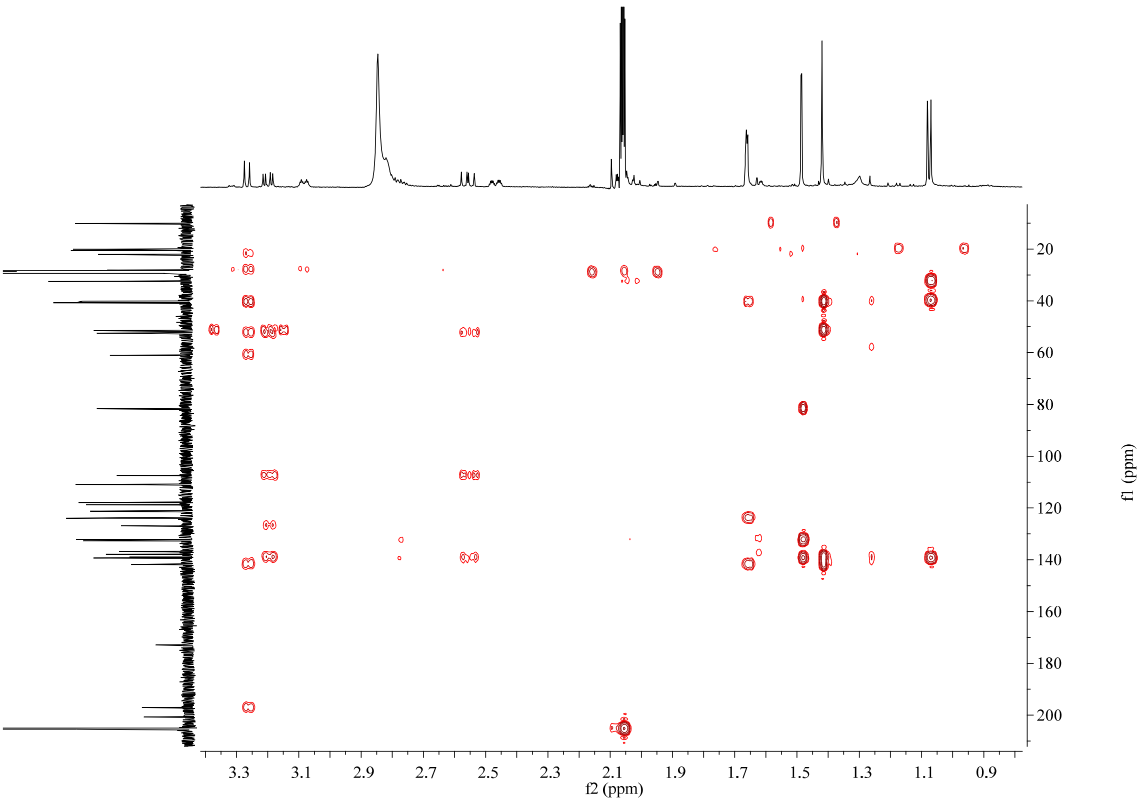

Figure S7. Expansion of HMBC spectrum (600/150 MHz) of compound **1** in CD_3COCD_3.

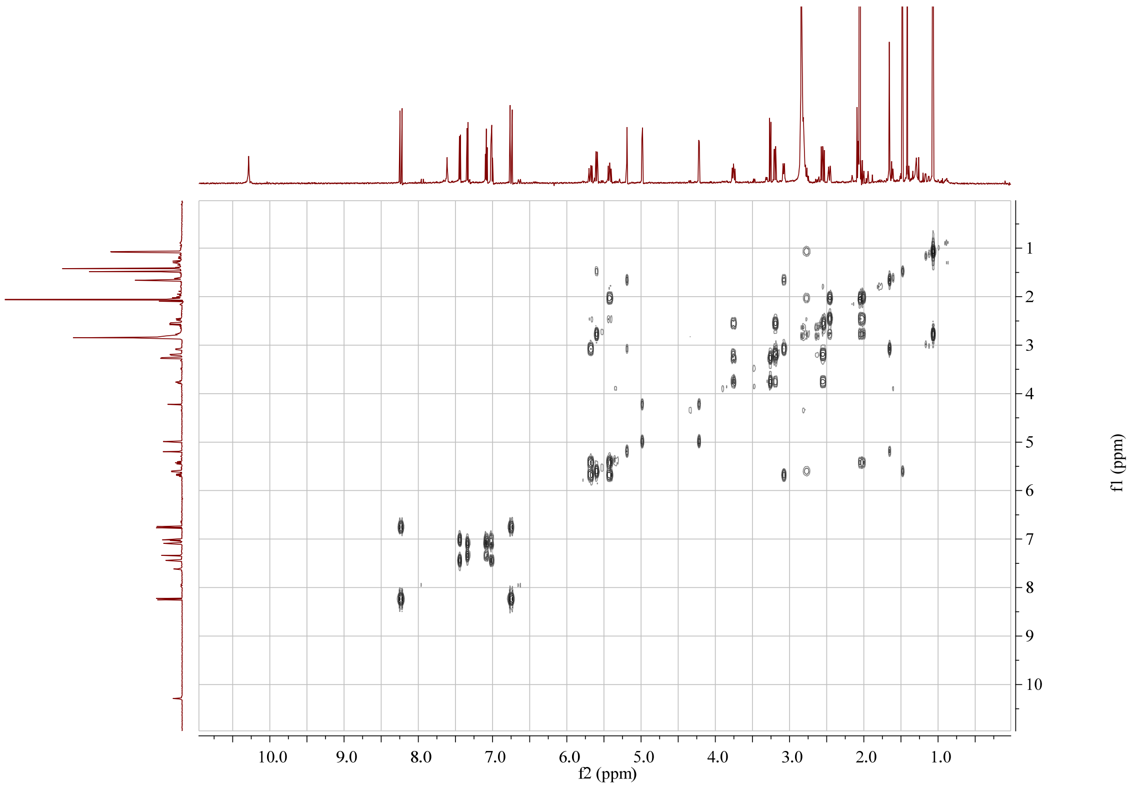

Figure S8. ^{1}H-^{1}HCOSY spectrum (600 MHz) of compound **1** in CD_3COCD_3.

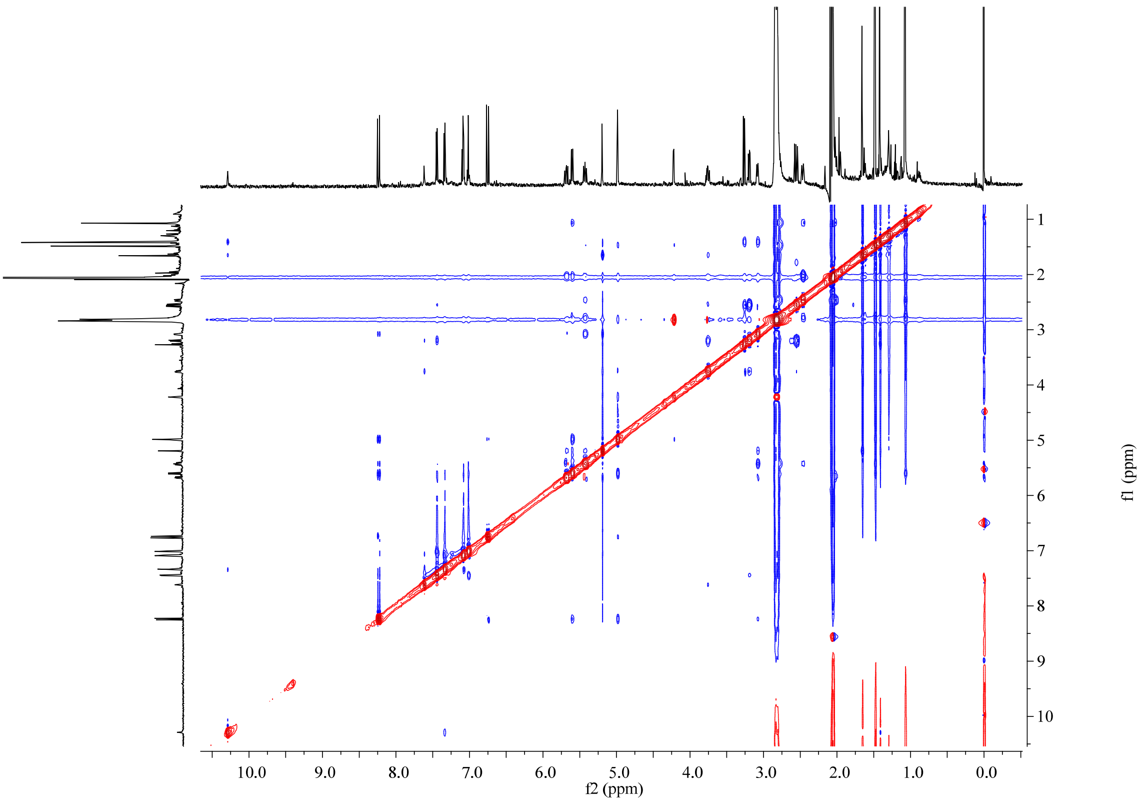

Figure S9. NOESY spectrum (600 MHz) of compound **1** in CD_3COCD_3.

Mass Spectrum SmartFormula Report

Analysis Info

Analysis Name	D:\Data\MS\data\201603\liwensheng_VH-2_41_01_1531.d	Acquisition Date	3/28/2016 4:15:36 PM
Method	LC_Direct Infusion_pos_100-1000mz.m	Operator	SCSIO
Sample Name	liwensheng_VH-2	Instrument / Ser#	maXis 29
Comment			

Acquisition Parameter

Source Type	ESI	Ion Polarity	Positive	Set Nebulizer	0.4 Bar
Focus	Active	Set Capillary	4500 V	Set Dry Heater	180 °C
Scan Begin	100 m/z	Set End Plate Offset	-500 V	Set Dry Gas	4.0 l/min
Scan End	2000 m/z	Set Collision Cell RF	800.0 Vpp	Set Divert Valve	Waste

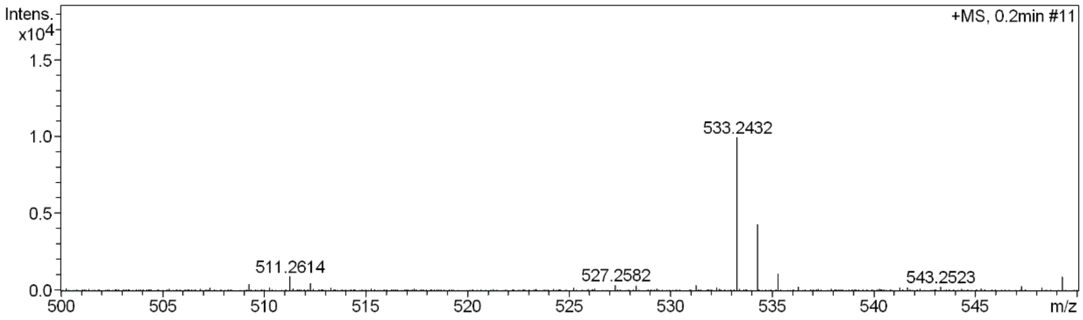

Meas. m/z	#	Formula	m/z	err [ppm]	err [mDa]	mSigma	rdb	e⁻ Conf	N-Rule
511.2614	1	C 32 H 35 N 2 O 4	511.2591	-4.4	-2.2	70.5	16.5	even	ok
533.2432	1	C 32 H 34 N 2 Na O 4	533.2411	-3.9	-2.1	41.1	16.5	even	ok

Figure S10. HRESIMS spectrum of compound **1**.

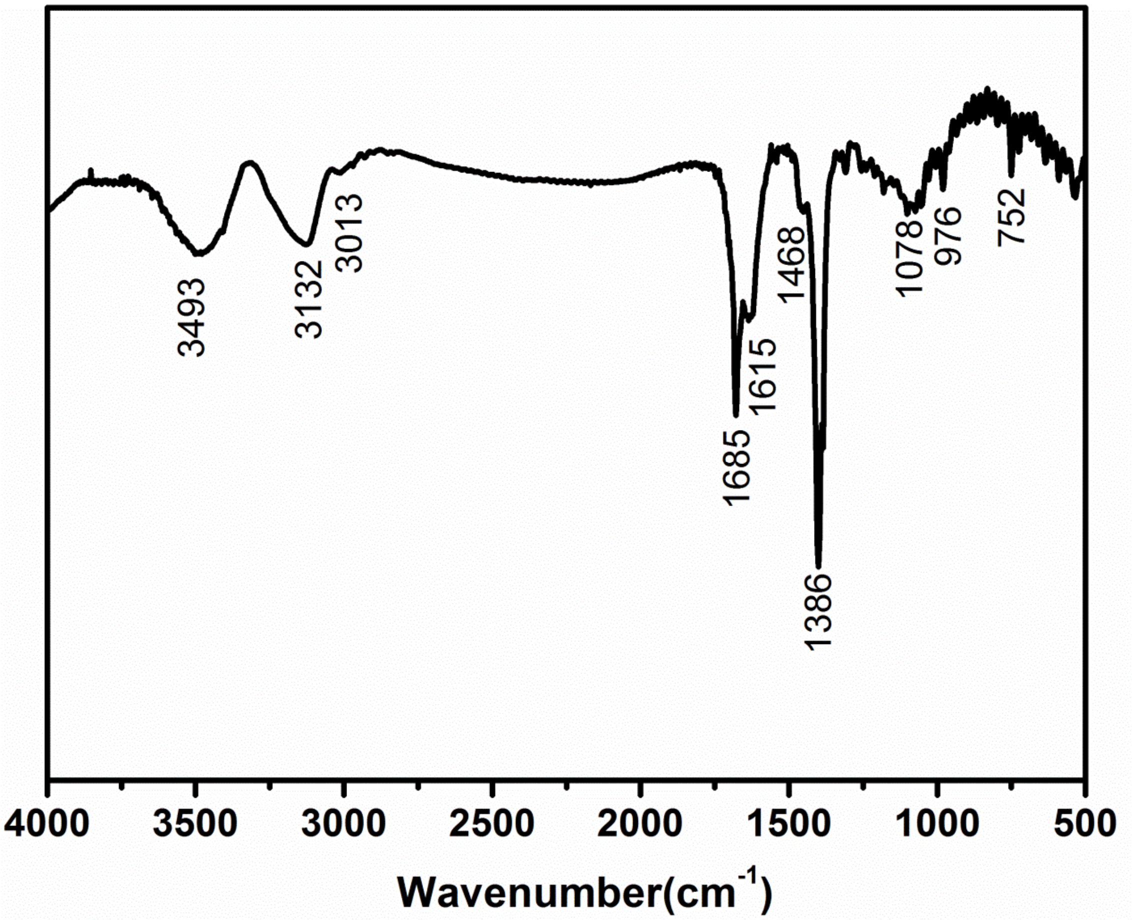

Figure S11. IR spectrum of compound **1**.

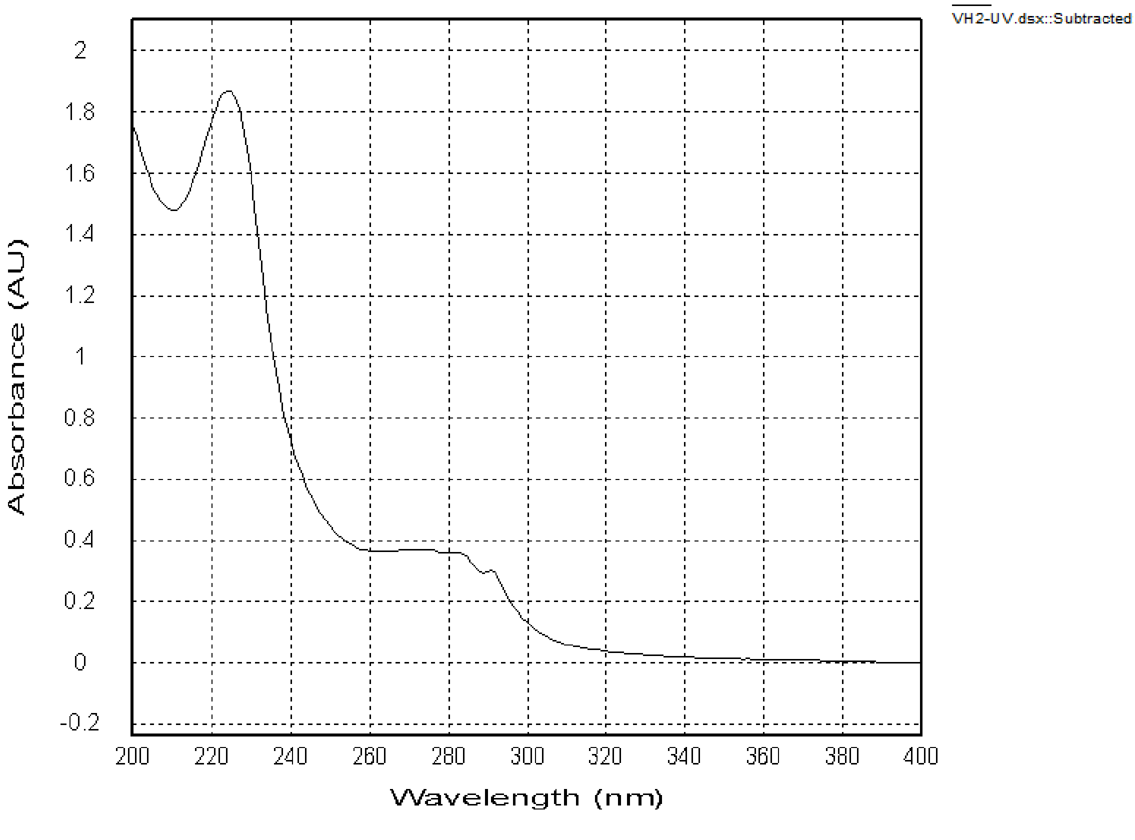

Figure S12. UV spectrum of compound **1**.

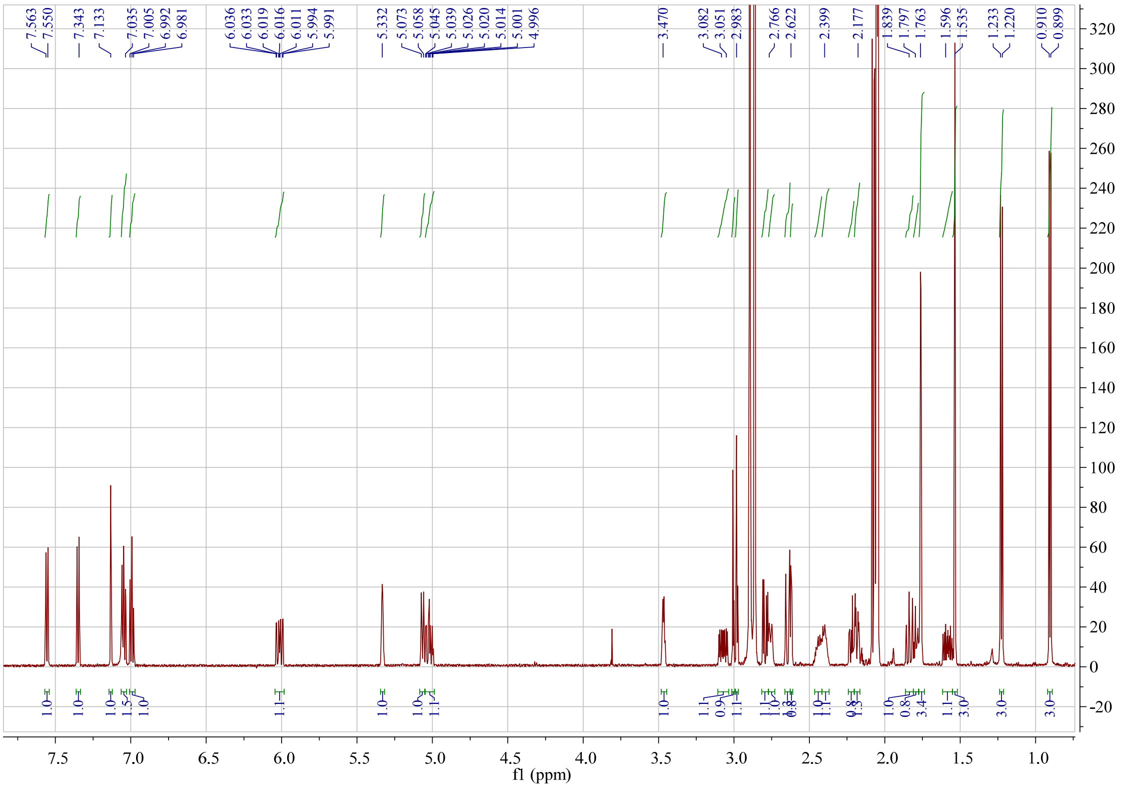

Figure S13. ^{1}H NMR spectrum (600 MHz) of compound **2** in CD_3COCD_3.

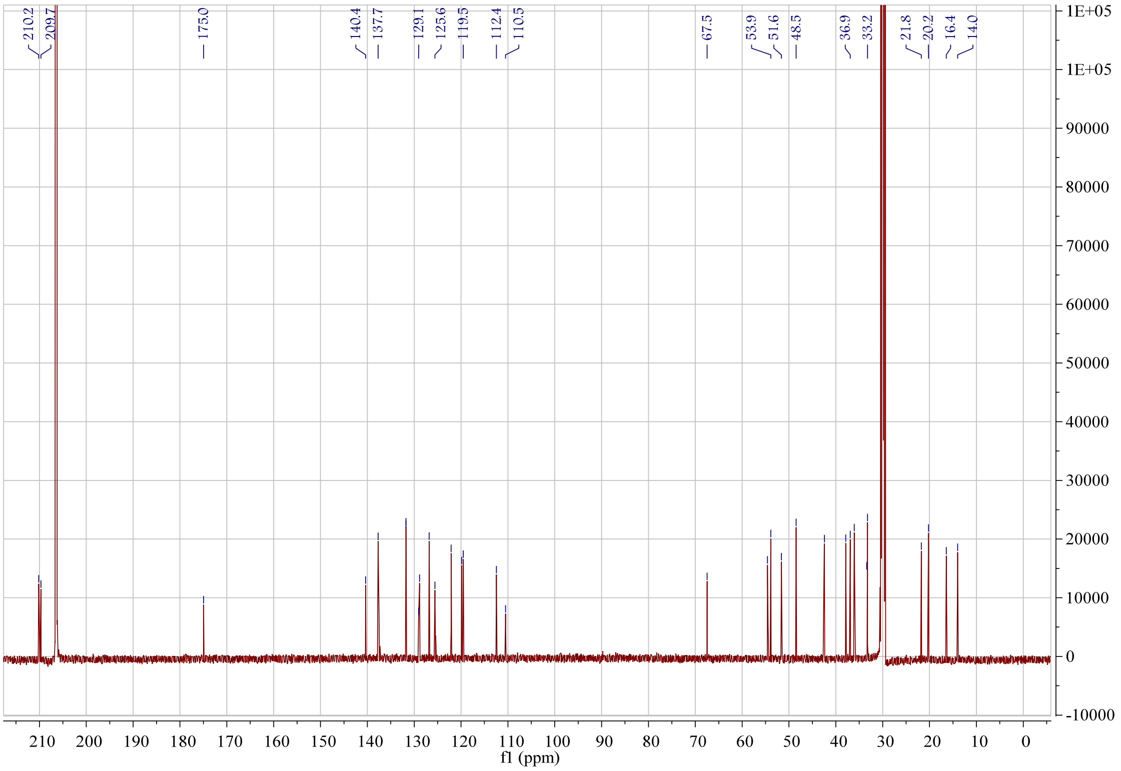

Figure S14. ^{13}C NMR spectrum (150 MHz) of compound **2** in CD_3COCD_3.

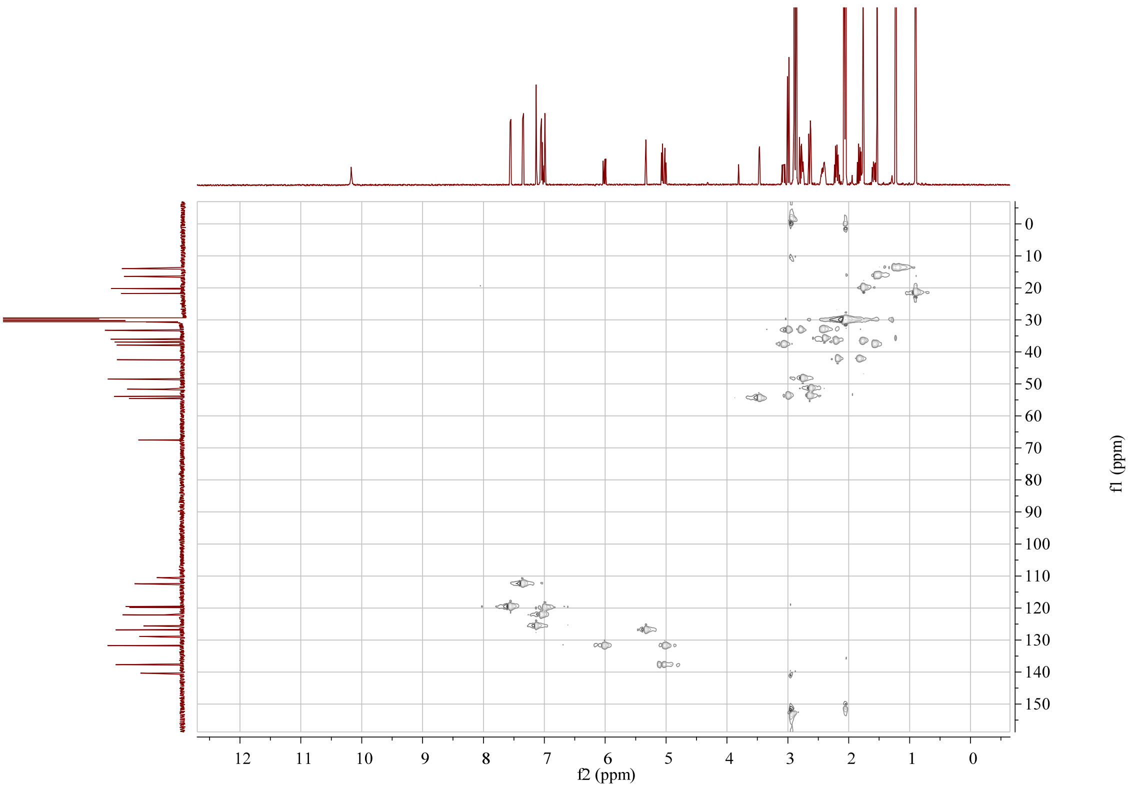

Figure S15. HSQC spectrum (600/150 MHz) of compound **2** in CD_3COCD_3.

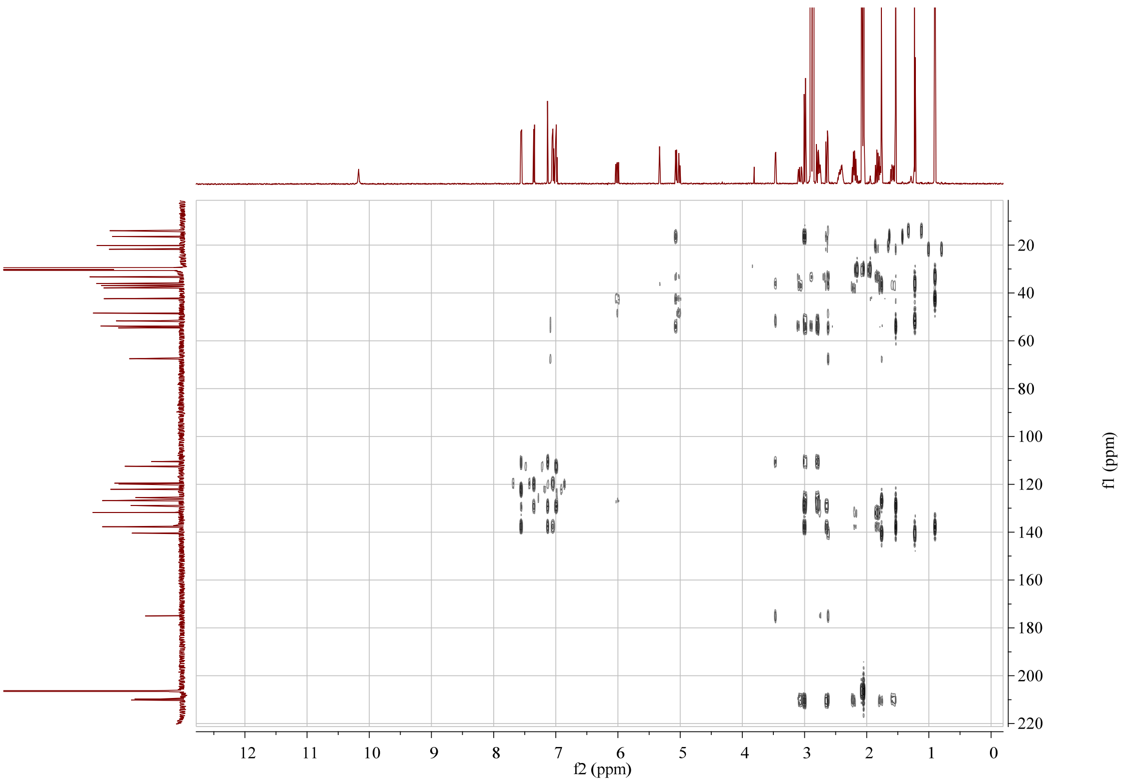

Figure S16. HMBC spectrum (600/150 MHz) of compound **2** in CD_3COCD_3.

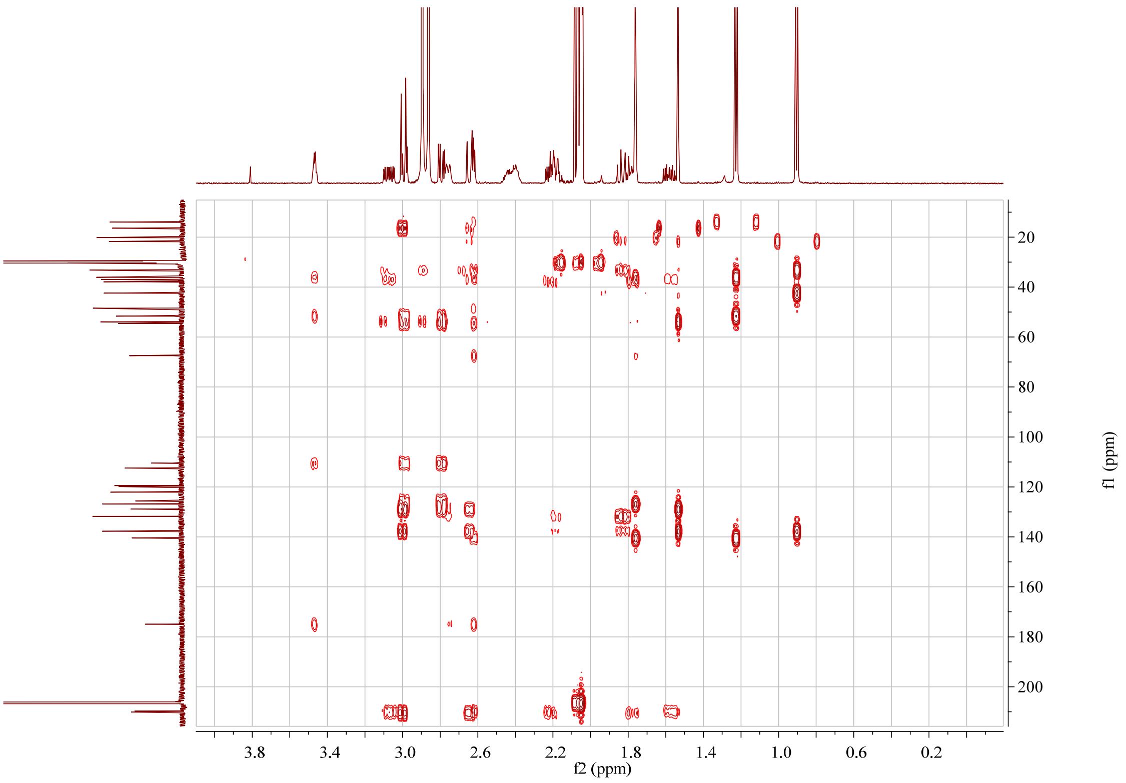

Figure S17. Expansion of HMBC spectrum (600/150 MHz) of compound **2** in CD_3COCD_3.

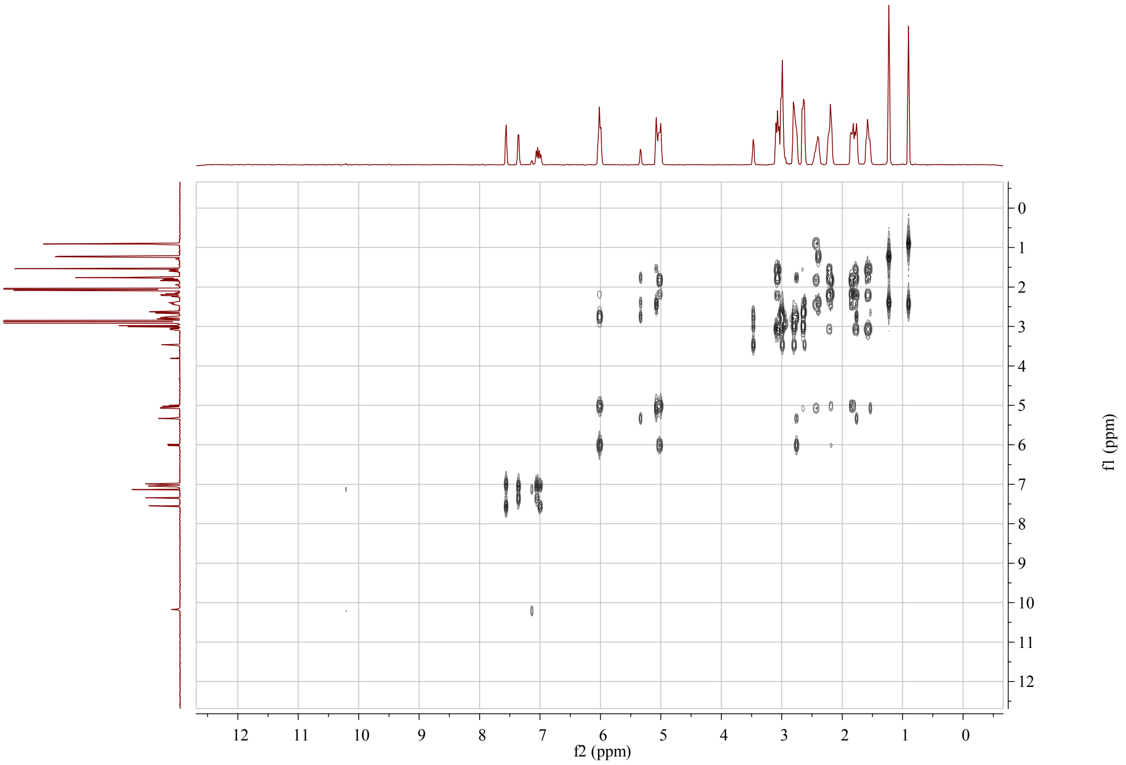

Figure S18. ^1H-^1HCOSY spectrum (600 MHz) of compound **2** in CD_3COCD_3.

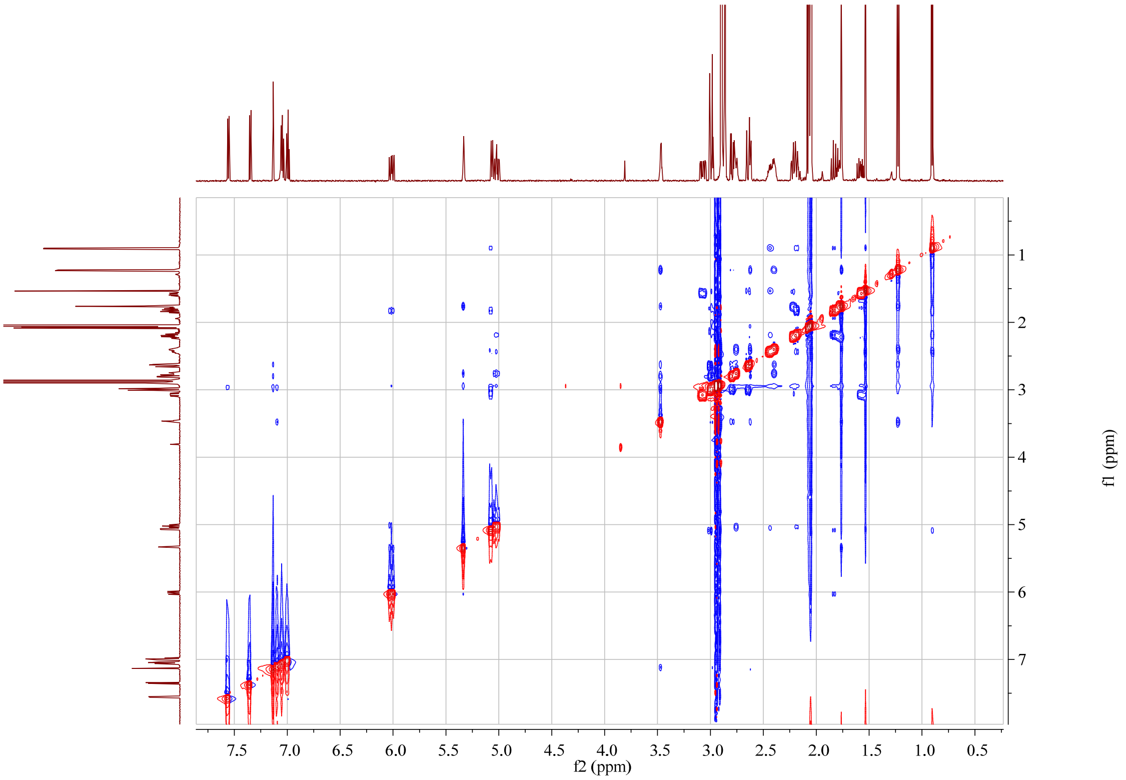

Figure S19. NOESY spectrum (600 MHz) of compound **2** in CD_3COCD_3.

Mass Spectrum SmartFormula Report

Analysis Info

Analysis Name	D:\Data\MS\data\201410\huanong_wangjinhua_J9_pos.d	Acquisition Date	10/27/2014 4:56:46 PM
Method	POS_100-2000_Dirrect Infusion.m	Operator	SCSIO
Sample Name	SCSIO	Instrument / Ser#	maXis 29
Comment			

Acquisition Parameter

Source Type	ESI	Ion Polarity	Positive	Set Nebulizer	0.3 Bar
Focus	Not active	Set Capillary	4500 V	Set Dry Heater	180 °C
Scan Begin	100 m/z	Set End Plate Offset	-500 V	Set Dry Gas	4.0 l/min
Scan End	2000 m/z	Set Collision Cell RF	2000.0 Vpp	Set Divert Valve	Waste

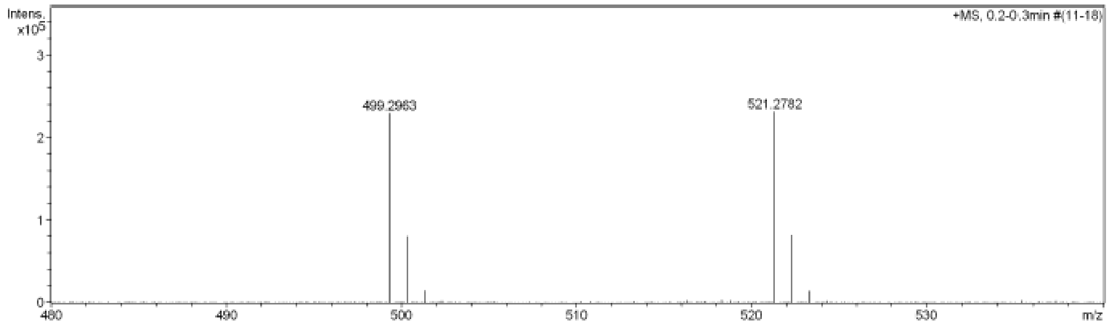

Meas. m/z	#	Formula	m/z	err [ppm]	Mean err [ppm]	rdb	N-Rule	e⁻ Conf	mSigma	Std I	Std Mean m/z	Std I VarNorm	Std m/z Diff	Std Comb Dev
499.2963	1	C 32 H 39 N 2 O 3	499.2955	-1.6	-1.5	14.5	ok	even	4.6	7.5	0.8	2.5	0.1	842.7
521.2782	1	C 32 H 38 N 2 Na O 3	521.2775	-1.4	-1.3	14.5	ok	even	5.1	9.3	0.7	3.3	0.3	807.3

Figure S20. HRESIMS spectrum of compound **2**.

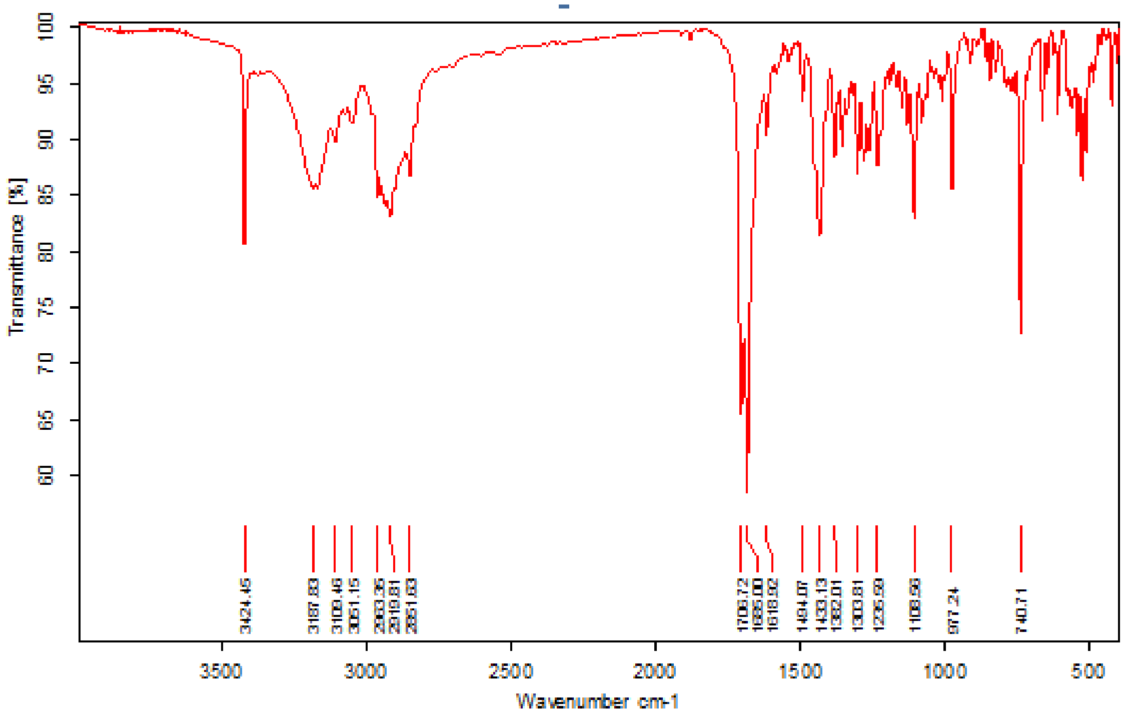

Figure S21. IR spectrum of compound **2**.

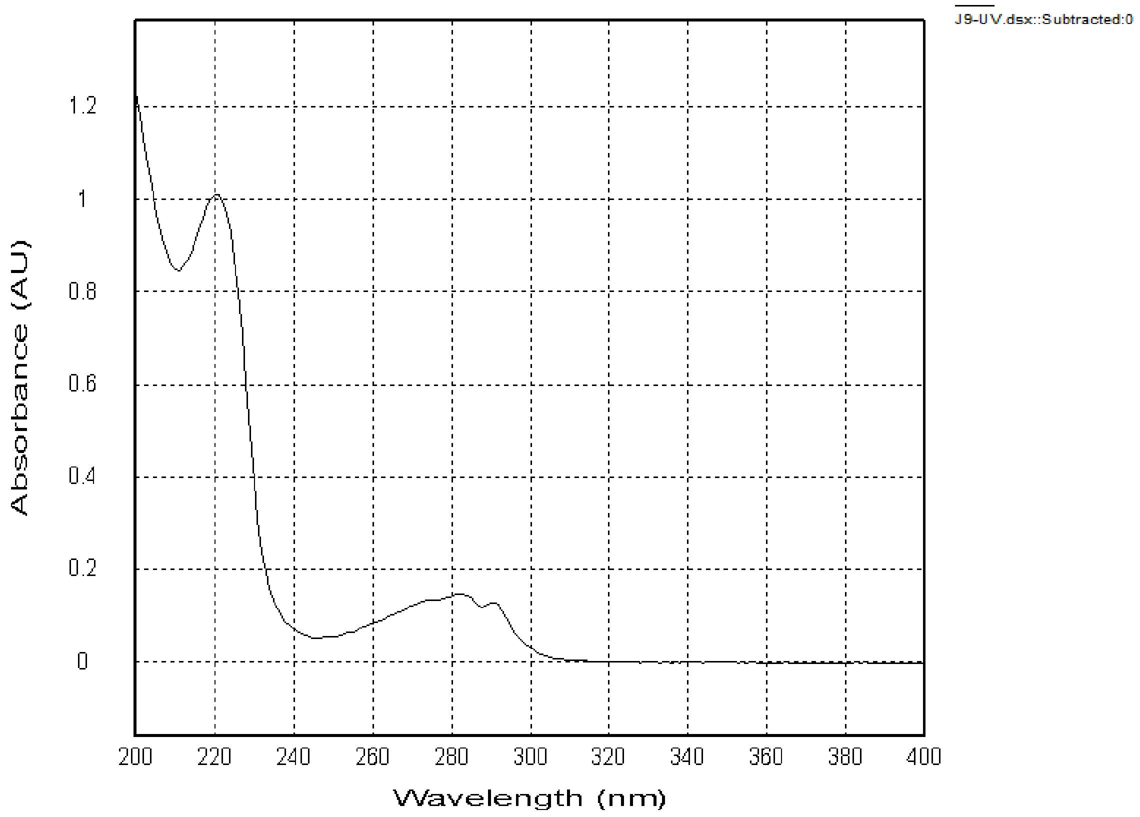

Figure S22. UV spectrum of compound **2**.

ECD Computational Details of Compounds 1 and 2

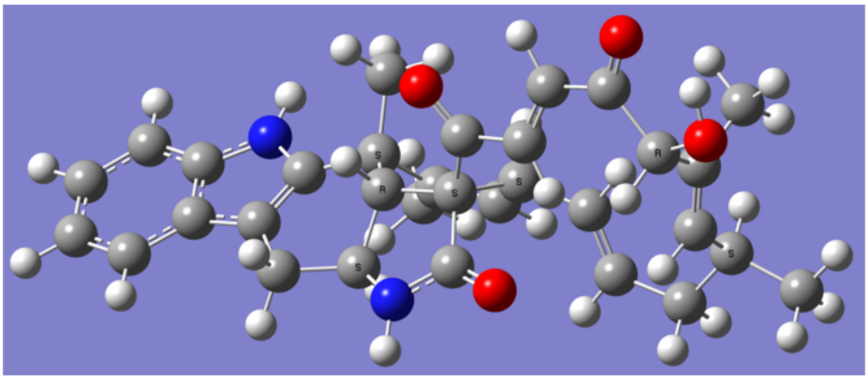

1a (0.0, **99.79%**)

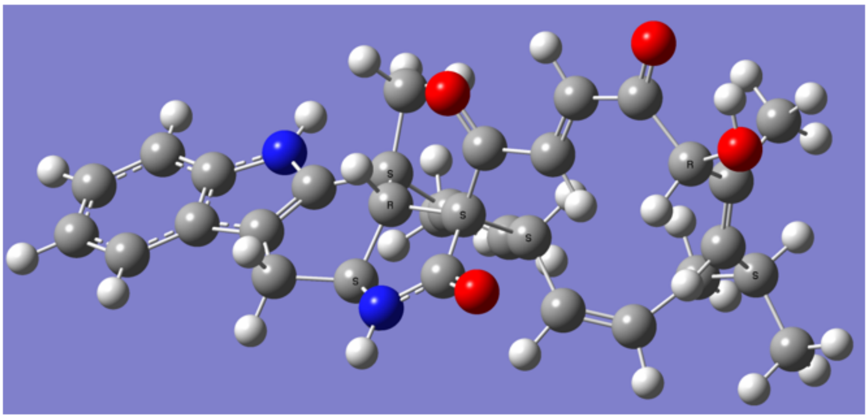

1b (3.76, **0.18%**)

Figure S23. Cartesian coordinate of optimized (3*S*,4*R*,5*S*,8*S*,9*S*,16*S*,19*R*)-**1**.

Table S1. Standard orientation of **1a**.

Center Number	Atomic Number	Atomic Type	Coordinates (Angstroms)		
			X	Y	Z
1	6	0	−0.279914	−0.450739	1.998085
2	7	0	0.935255	−0.280169	2.568889
3	6	0	1.990761	0.069606	1.627701
4	6	0	1.429194	−0.506881	0.307318
5	6	0	2.164752	0.088001	−0.917356
6	6	0	1.683173	1.525009	−1.152016
7	6	0	0.449189	1.895377	−0.786431
8	6	0	−0.628308	1.072192	−0.099204
9	6	0	−0.097751	−0.321711	0.448492
10	6	0	3.378671	−0.491203	1.921469
11	6	0	1.889143	−0.737866	−2.202252
12	6	0	2.592807	2.497185	−1.861414
13	6	0	−1.307497	1.944601	0.943632
14	6	0	−2.429373	2.662014	0.794712
15	6	0	−3.323007	2.765447	−0.413273
16	6	0	−4.827924	2.440593	−0.161208
17	6	0	−4.994595	1.016486	0.305197
18	6	0	−5.442130	−0.039255	−0.391168
19	6	0	−5.487702	−1.407703	0.284895
20	6	0	−4.642043	−2.453707	−0.470330

21	6	0	−3.172751	−2.357719	−0.585515
22	6	0	−2.396271	−1.410885	−0.032767
23	6	0	−0.905787	−1.472395	−0.176958
24	6	0	−5.483579	3.422642	0.826822
25	6	0	−5.950277	−0.017314	−1.810620
26	7	0	4.667341	0.098536	−1.493610
27	6	0	3.644414	−0.011249	−0.562875
28	6	0	4.194156	−0.286748	0.673740
29	6	0	5.621264	−0.341246	0.505149
30	6	0	6.704312	−0.573842	1.372195
31	6	0	7.996127	−0.553082	0.857950
32	6	0	8.231684	−0.305117	−0.511046
33	6	0	7.181161	−0.072080	−1.393523
34	6	0	5.882971	−0.093811	−0.872080
35	8	0	−1.333410	−0.682386	2.582558
36	8	0	−0.367105	−2.411883	−0.741878
37	8	0	−5.225811	−3.410093	−0.976284
38	8	0	−6.817591	−1.887826	0.362875
39	1	0	1.619833	−1.585354	0.319332
40	1	0	3.832196	0.019566	2.779712
41	1	0	3.303601	−1.555240	2.187143
42	1	0	2.073781	1.161736	1.549623
43	1	0	−1.373017	0.845870	−0.874410
44	1	0	0.124356	2.907615	−1.020421
45	1	0	4.548859	0.289479	−2.475617
46	1	0	7.361288	0.118546	−2.447303
47	1	0	9.251722	−0.296058	−0.883749
48	1	0	8.840264	−0.730716	1.517958
49	1	0	6.534550	−0.766546	2.428003
50	1	0	1.028528	−0.209787	3.573689
51	1	0	−0.766842	2.050466	1.882411
52	1	0	−2.723610	3.280863	1.641571
53	1	0	−3.271650	3.797967	−0.789207
54	1	0	−2.963919	2.125226	−1.224563
55	1	0	−5.323182	2.566364	−1.129865
56	1	0	−2.806478	−0.598379	0.550423
57	1	0	−4.677377	0.840606	1.334757
58	1	0	−2.712663	−3.162895	−1.152728
59	1	0	−5.070491	−1.316075	1.298023
60	1	0	−5.334048	4.458749	0.504615
61	1	0	−5.070877	3.319921	1.835869
62	1	0	−6.560591	3.240390	0.892792
63	1	0	−5.246724	−0.510910	−2.493819
64	1	0	−6.895525	−0.565830	−1.872093
65	1	0	−6.116184	0.994319	−2.183221
66	1	0	−6.819347	−2.708562	−0.166232
67	1	0	2.097078	3.460804	−2.002856
68	1	0	3.516782	2.666759	−1.298079
69	1	0	2.889127	2.130442	−2.852217
70	1	0	0.822512	−0.750469	−2.431066
71	1	0	2.404256	−0.307206	−3.066503
72	1	0	2.227720	−1.770325	−2.081040

Table S2. Standard orientation of **1b**.

Center Number	Atomic Number	Atomic Type	Coordinates (Angstroms)		
			X	Y	Z
1	6	0	−0.371849	0.094316	1.776503
2	7	0	0.824033	0.219563	2.407457
3	6	0	1.952702	0.327790	1.500322
4	6	0	1.444082	−0.427715	0.248719
5	6	0	2.242409	0.076256	−0.984726
6	6	0	1.791920	1.520405	−1.274056
7	6	0	0.498482	1.834924	−1.112837
8	6	0	−0.591184	0.831556	−0.787172
9	6	0	−0.095277	−0.260325	0.277433
10	6	0	3.293718	−0.239236	1.954170
11	6	0	2.011015	−0.810377	−2.234159
12	6	0	2.798664	2.542519	−1.731814
13	6	0	−1.947537	1.441647	−0.503411
14	6	0	−2.197207	2.540286	0.211930
15	6	0	−3.559906	3.100680	0.533729
16	6	0	−4.796216	2.375025	−0.043322
17	6	0	−4.911291	0.952577	0.465044
18	6	0	−5.331736	−0.128473	−0.210222
19	6	0	−5.384203	−1.483213	0.492212
20	6	0	−4.577629	−2.564378	−0.264097
21	6	0	−3.111292	−2.487087	−0.435834
22	6	0	−2.317508	−1.589291	0.169504
23	6	0	−0.837645	−1.572016	−0.058893
24	6	0	−6.071674	3.169412	0.310084
25	6	0	−5.815641	−0.134749	−1.639187
26	7	0	4.768363	−0.043641	−1.432965
27	6	0	3.699933	−0.035982	−0.549203
28	6	0	4.179996	−0.204211	0.735906
29	6	0	5.610667	−0.315368	0.644984
30	6	0	6.642977	−0.490955	1.584406
31	6	0	7.957617	−0.559462	1.136850
32	6	0	8.266150	−0.456935	−0.236356
33	6	0	7.267357	−0.281733	−1.189509
34	6	0	5.945778	−0.212258	−0.735096
35	8	0	−1.466184	0.183017	2.324459
36	8	0	−0.257004	−2.538285	−0.530158
37	8	0	−5.193934	−3.517488	−0.735402
38	8	0	−6.721452	−1.937560	0.607649
39	1	0	1.661722	−1.492212	0.382001
40	1	0	3.166245	−1.260478	2.339940
41	1	0	3.718390	0.360015	2.769154
42	1	0	2.111384	1.384069	1.249466
43	1	0	−0.730337	0.244065	−1.706261
44	1	0	0.163294	2.846918	−1.326344
45	1	0	4.699920	0.028702	−2.435605
46	1	0	7.503972	−0.201777	−2.246428
47	1	0	9.302414	−0.515162	−0.556118
48	1	0	8.763007	−0.694496	1.852988
49	1	0	6.416349	−0.571807	2.644012

50	1	0	0.844235	0.582741	3.352046
51	1	0	−2.785419	0.892740	−0.919163
52	1	0	−1.362825	3.094862	0.644431
53	1	0	−3.593243	4.150851	0.209863
54	1	0	−3.656628	3.143150	1.630100
55	1	0	−4.699416	2.363377	−1.135584
56	1	0	−2.705618	−0.831781	0.837157
57	1	0	−4.622191	0.817144	1.508785
58	1	0	−2.687327	−3.242562	−1.092800
59	1	0	−4.944280	−1.380444	1.494823
60	1	0	−6.957104	2.706342	−0.134978
61	1	0	−6.003765	4.200227	−0.054441
62	1	0	−6.221797	3.204720	1.395105
63	1	0	−6.773093	−0.662465	−1.700564
64	1	0	−5.956058	0.869673	−2.040378
65	1	0	−5.115892	−0.664029	−2.299679
66	1	0	−6.748042	−2.770057	0.097826
67	1	0	3.598341	2.679430	−0.995288
68	1	0	3.283861	2.238752	−2.667876
69	1	0	2.319389	3.509601	−1.904861
70	1	0	2.571665	−0.431144	−3.094363
71	1	0	2.328459	−1.838869	−2.041636
72	1	0	0.958186	−0.830808	−2.520557

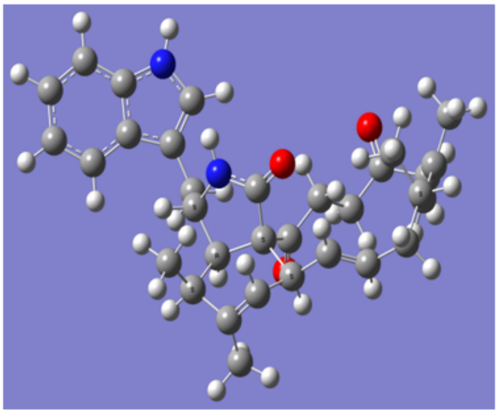

2a (0.62, **21.59%)**

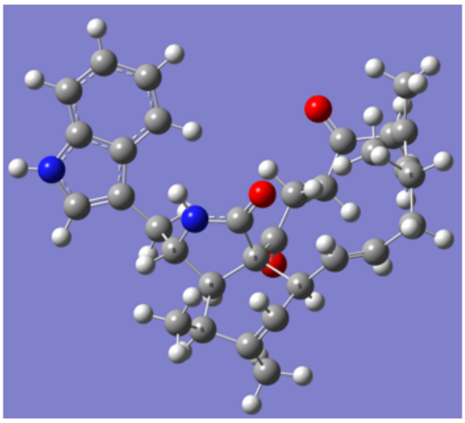

2b (1.12, **9.20%)**

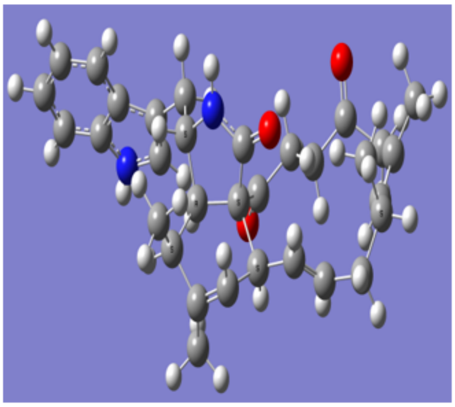

2c (1.22, **7.85%)**

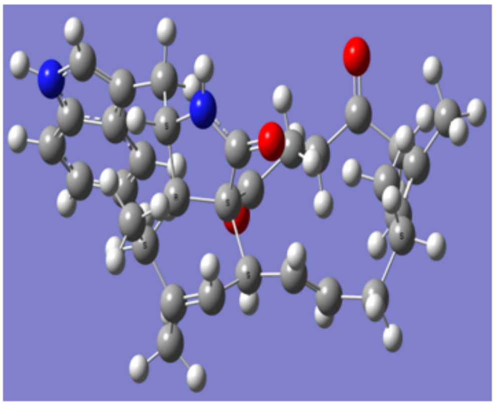

2d (0.0, **61.35%)**

Figure S24. Cartesian coordinate of optimized (3*S*,4*R*,5*S*,8*S*,9*S*,16*S*)-**2**.

Table S3. Standard orientation of **2a**.

Center Number	Atomic Number	Atomic Type	Coordinates (Angstroms)		
			X	Y	Z
1	6	0	−0.327673	0.772108	−1.057831
2	7	0	−1.685698	0.800002	−1.034391
3	6	0	−2.286669	1.510116	0.088998
4	6	0	−1.121173	2.425640	0.554174
5	6	0	−1.303312	3.963875	0.022042
6	6	0	0.135616	4.383342	0.098375
7	6	0	0.900322	3.723589	−0.812469
8	6	0	1.579165	2.526876	−0.234812
9	6	0	0.178870	1.618247	0.124988
10	6	0	−2.844309	0.565118	1.184144
11	6	0	−2.007253	4.140386	−1.332065
12	6	0	0.650179	4.810481	1.448685
13	6	0	2.606336	1.839795	−1.086980
14	6	0	3.803512	1.459480	−0.623159
15	6	0	4.902960	0.787619	−1.415513
16	6	0	4.798046	−0.770375	−1.463393
17	6	0	4.613813	−1.323511	−0.058112
18	6	0	4.432288	−2.590021	0.352857
19	6	0	4.046275	−2.844244	1.812838
20	6	0	2.535728	−2.623983	1.905569
21	6	0	2.035953	−1.207487	2.185580
22	6	0	1.210928	−0.646996	1.018644
23	6	0	0.644667	0.732014	1.307620
24	6	0	3.680534	−1.184272	−2.442984
25	6	0	4.471051	−3.822344	−0.512929
26	7	0	−6.149267	−0.899350	0.336332
27	6	0	−5.342123	0.063149	0.905037
28	6	0	−4.016319	−0.260335	0.729061
29	6	0	−4.001906	−1.511459	0.004781
30	6	0	−2.986386	−2.367568	−0.464741
31	6	0	−3.336786	−3.535383	−1.132227
32	6	0	−4.689405	−3.875079	−1.347404
33	6	0	−5.717276	−3.055030	−0.895295
34	6	0	−5.360354	−1.880791	−0.221661
35	8	0	0.345054	0.151411	−1.879025
36	8	0	1.746258	−3.534289	1.700915
37	8	0	0.615590	1.176589	2.445334
38	1	0	−1.120384	2.492422	1.641729
39	1	0	−2.040511	−0.082213	1.553733
40	1	0	−3.151780	1.194453	2.027289
41	1	0	−3.129828	2.103534	−0.272157
42	1	0	−1.917703	4.466684	0.779744
43	1	0	2.009134	2.738356	0.746510
44	1	0	0.427475	3.493829	−1.769205
45	1	0	−2.218370	0.208428	−1.658352
46	1	0	2.355807	1.702720	−2.135311
47	1	0	4.009593	1.623379	0.435513
48	1	0	5.870045	1.062681	−0.977822
49	1	0	4.904936	1.157798	−2.447054

50	1	0	5.750596	−1.138154	−1.873277
51	1	0	0.373679	−1.322497	0.797648
52	1	0	1.801084	−0.600761	0.101059
53	1	0	4.587506	−0.568813	0.725424
54	1	0	2.868126	−0.536456	2.408954
55	1	0	1.410893	−1.253619	3.084129
56	1	0	4.572456	−2.159348	2.483390
57	1	0	4.269533	−3.873945	2.104600
58	1	0	3.581814	−2.268444	−2.523267
59	1	0	3.903430	−0.798026	−3.444485
60	1	0	2.712454	−0.780427	−2.135395
61	1	0	5.127862	−4.575562	−0.060775
62	1	0	3.476914	−4.281443	−0.587439
63	1	0	4.836057	−3.620134	−1.520475
64	1	0	−1.936950	5.188378	−1.637952
65	1	0	−3.069800	3.897574	−1.259126
66	1	0	−1.582929	3.525750	−2.128341
67	1	0	1.740035	4.891245	1.462527
68	1	0	0.238285	5.796553	1.699401
69	1	0	0.343734	4.131236	2.255039
70	1	0	−2.557902	−4.199254	−1.495671
71	1	0	−4.932108	−4.793468	−1.873713
72	1	0	−6.759304	−3.314449	−1.056876
73	1	0	−1.940111	−2.122257	−0.308561
74	1	0	−7.157308	−0.893638	0.350151
75	1	0	−5.778696	0.915898	1.406132

Table S4. Standard orientation of **2b**.

Center Number	Atomic Number	Atomic Type	Coordinates (Angstroms)		
			X	Y	Z
1	6	0	−0.366223	0.879793	1.557769
2	7	0	0.840308	0.591437	2.111108
3	6	0	1.942383	0.412593	1.171638
4	6	0	1.414229	1.149477	−0.087193
5	6	0	2.038805	2.652783	−0.233463
6	6	0	0.969745	3.235795	−1.112575
7	6	0	−0.217453	3.349073	−0.458121
8	6	0	−1.119778	2.184115	−0.703713
9	6	0	−0.161681	0.990337	0.034521
10	6	0	2.290261	−1.084704	0.957388
11	6	0	2.350492	3.401458	1.070745
12	6	0	1.069318	2.948865	−2.588692
13	6	0	−2.533528	2.289662	−0.208074
14	6	0	−3.594265	1.921249	−0.937596
15	6	0	−5.045747	2.012901	−0.521174
16	6	0	−5.565786	0.772906	0.273348
17	6	0	−5.218620	−0.509112	−0.467940
18	6	0	−5.453668	−1.787523	−0.126689
19	6	0	−4.797649	−2.904270	−0.943479
20	6	0	−3.365558	−3.040384	−0.423987
21	6	0	−2.284847	−2.173266	−1.068137
22	6	0	−1.694632	−1.157758	−0.079729
23	6	0	−0.599940	−0.305332	−0.693890

24	6	0	−5.021917	0.813641	1.716780
25	6	0	−6.238808	−2.258276	1.069763
26	7	0	4.715173	−1.754603	−1.775118
27	6	0	3.434058	−1.664611	−1.271135
28	6	0	3.470671	−1.305593	0.056590
29	6	0	4.866768	−1.162538	0.397071
30	6	0	5.558051	−0.829452	1.577115
31	6	0	6.947416	−0.789533	1.558787
32	6	0	7.669399	−1.077538	0.380618
33	6	0	7.017140	−1.414119	−0.800835
34	6	0	5.617815	−1.453098	−0.778383
35	8	0	−1.415662	1.000257	2.186962
36	8	0	−3.097054	−3.773331	0.516453
37	8	0	−0.110777	−0.585907	−1.778393
38	1	0	1.735886	0.615565	−0.979991
39	1	0	2.487187	−1.519845	1.945756
40	1	0	1.415116	−1.609361	0.560763
41	1	0	2.836929	0.902657	1.563190
42	1	0	2.987008	2.523826	−0.770929
43	1	0	−1.128914	1.893523	−1.756481
44	1	0	−0.163959	3.595060	0.604157
45	1	0	0.911256	0.375931	3.096804
46	1	0	−2.672123	2.730440	0.775271
47	1	0	−3.404881	1.487432	−1.920506
48	1	0	−5.662261	2.140230	−1.418952
49	1	0	−5.203657	2.901507	0.100647
50	1	0	−6.660047	0.871483	0.330028
51	1	0	−1.279165	−1.677287	0.793871
52	1	0	−2.466700	−0.493409	0.315481
53	1	0	−4.663493	−0.363916	−1.392573
54	1	0	−2.671695	−1.654180	−1.947647
55	1	0	−1.492440	−2.845116	−1.415851
56	1	0	−4.793644	−2.660938	−2.009365
57	1	0	−5.316908	−3.854831	−0.794780
58	1	0	−5.382701	−0.024646	2.315955
59	1	0	−5.346998	1.736627	2.211014
60	1	0	−3.928983	0.791710	1.731813
61	1	0	−6.752662	−1.446178	1.585470
62	1	0	−6.993096	−2.990317	0.755883
63	1	0	−5.588244	−2.771903	1.789199
64	1	0	2.663747	4.421614	0.829708
65	1	0	3.176137	2.930801	1.609630
66	1	0	1.502164	3.461577	1.755430
67	1	0	0.149459	3.211397	−3.117409
68	1	0	1.884101	3.544924	−3.019846
69	1	0	1.305364	1.898550	−2.805174
70	1	0	7.489687	−0.533450	2.464337
71	1	0	8.754569	−1.037628	0.397010
72	1	0	7.571824	−1.638810	−1.707080
73	1	0	5.014872	−0.608550	2.492159
74	1	0	4.949090	−2.009629	−2.721914
75	1	0	2.579062	−1.866086	−1.901164

Table S5. Standard orientation of **2c**.

Center Number	Atomic Number	Atomic Type	Coordinates (Angstroms)		
			X	Y	Z
1	6	0	0.469950	1.320463	−1.575139
2	7	0	−0.641211	1.199002	−2.344130
3	6	0	−1.887093	0.920851	−1.638841
4	6	0	−1.535214	1.333620	−0.182361
5	6	0	−2.139080	2.805454	0.202273
6	6	0	−1.199878	3.129977	1.326713
7	6	0	0.073005	3.318488	0.884743
8	6	0	0.902647	2.082063	0.997022
9	6	0	0.040688	1.121004	−0.109398
10	6	0	−2.361301	−0.545204	−1.825293
11	6	0	−2.233514	3.827954	−0.940408
12	6	0	−1.521178	2.537464	2.674722
13	6	0	2.376663	2.208054	0.739290
14	6	0	3.306678	1.630751	1.510597
15	6	0	4.805721	1.719481	1.326371
16	6	0	5.398376	0.651102	0.353420
17	6	0	4.900901	−0.736759	0.726738
18	6	0	5.171088	−1.922894	0.156169
19	6	0	4.370554	−3.155209	0.588530
20	6	0	3.032452	−3.093329	−0.149628
21	6	0	1.883277	−2.337758	0.516636
22	6	0	1.464638	−1.098369	−0.286590
23	6	0	0.346136	−0.316590	0.376657
24	6	0	5.081694	1.040217	−1.105900
25	6	0	6.143368	−2.163961	−0.968853
26	7	0	−6.026310	−0.815030	−1.288176
27	6	0	−4.915958	−0.476642	−2.032303
28	6	0	−3.766218	−0.788000	−1.342062
29	6	0	−4.195168	−1.363349	−0.087679
30	6	0	−3.524468	−1.892474	1.032666
31	6	0	−4.271401	−2.393614	2.092564
32	6	0	−5.682559	−2.381602	2.064900
33	6	0	−6.375364	−1.869177	0.973835
34	6	0	−5.620743	−1.365964	−0.092834
35	8	0	1.603158	1.523367	−2.007189
36	8	0	2.896772	−3.579693	−1.262553
37	8	0	−0.266015	−0.772002	1.332009
38	1	0	−1.999298	0.640055	0.517052
39	1	0	−2.299916	−0.768346	−2.897471
40	1	0	−1.664700	−1.229882	−1.329809
41	1	0	−2.676953	1.557706	−2.043936
42	1	0	−3.159398	2.617612	0.560064
43	1	0	0.747052	1.575225	1.951557
44	1	0	0.183523	3.788798	−0.094423
45	1	0	−0.569319	1.213949	−3.352942
46	1	0	2.672297	2.837212	−0.095603
47	1	0	2.962350	1.012302	2.340753
48	1	0	5.287622	1.611176	2.305346
49	1	0	5.080151	2.710211	0.946188

50	1	0	6.491286	0.686064	0.473720
51	1	0	1.137221	−1.389602	−1.293175
52	1	0	2.308674	−0.421171	−0.439643
53	1	0	4.192692	−0.760974	1.552122
54	1	0	2.145280	−2.048051	1.536276
55	1	0	1.037012	−3.029927	0.588686
56	1	0	4.208705	−3.159263	1.669887
57	1	0	4.886519	−4.075259	0.301200
58	1	0	5.495373	0.324868	−1.819848
59	1	0	5.515119	2.021543	−1.331879
60	1	0	4.003874	1.101286	−1.277348
61	1	0	6.741457	−1.283538	−1.207787
62	1	0	6.829453	−2.977307	−0.702098
63	1	0	5.618796	−2.485874	−1.877665
64	1	0	−2.516298	4.800610	−0.527124
65	1	0	−3.007248	3.546837	−1.658555
66	1	0	−1.300653	3.954160	−1.493459
67	1	0	−0.687933	2.642495	3.374188
68	1	0	−2.386090	3.059509	3.103999
69	1	0	−1.794979	1.475407	2.620620
70	1	0	−3.761941	−2.802352	2.960537
71	1	0	−6.237030	−2.780566	2.909268
72	1	0	−7.461021	−1.860157	0.946839
73	1	0	−2.439659	−1.903319	1.074545
74	1	0	−6.982172	−0.698494	−1.586519
75	1	0	−5.030727	−0.040422	−3.014901

Table S6. Standard orientation of **2d**.

Center Number	Atomic Number	Atomic Type	Coordinates (Angstroms)		
			X	Y	Z
1	6	0	−0.064990	0.605630	−1.112642
2	7	0	−1.408189	0.409033	−1.126684
3	6	0	−2.134977	0.896790	0.039332
4	6	0	−1.136601	1.924043	0.639791
5	6	0	−1.530668	3.465929	0.259095
6	6	0	−0.173995	4.081169	0.446651
7	6	0	0.712442	3.642888	−0.487624
8	6	0	1.537457	2.496683	−0.001109
9	6	0	0.280247	1.367817	0.180344
10	6	0	−2.549376	−0.240054	1.007450
11	6	0	−2.210014	3.682094	−1.101480
12	6	0	0.225127	4.431611	1.857085
13	6	0	2.689726	2.058830	−0.859293
14	6	0	3.902129	1.775616	−0.366478
15	6	0	5.116790	1.344116	−1.158654
16	6	0	5.209192	−0.195224	−1.406779
17	6	0	5.025616	−0.949851	−0.098781
18	6	0	5.015822	−2.273604	0.132397
19	6	0	4.589709	−2.782033	1.513035
20	6	0	3.060208	−2.774389	1.533125
21	6	0	2.355838	−1.494665	1.981522
22	6	0	1.549328	−0.853370	0.843548
23	6	0	0.828972	0.411871	1.270895

24	6	0	4.201609	−0.609351	−2.499323
25	6	0	5.295065	−3.353028	−0.880731
26	7	0	−4.233737	−3.105397	−0.653971
27	6	0	−3.125596	−2.480206	−0.119606
28	6	0	−3.470884	−1.244507	0.377091
29	6	0	−4.886431	−1.093570	0.131959
30	6	0	−5.824100	−0.079540	0.403156
31	6	0	−7.149947	−0.267102	0.029987
32	6	0	−7.565363	−1.452980	−0.612855
33	6	0	−6.664809	−2.475335	−0.892475
34	6	0	−5.330392	−2.282716	−0.514993
35	8	0	0.710991	0.208563	−1.979887
36	8	0	2.414733	−3.737763	1.147069
37	8	0	0.734907	0.721346	2.448936
38	1	0	−1.172118	1.873406	1.727856
39	1	0	−1.654187	−0.743739	1.386774
40	1	0	−3.035102	0.227950	1.873200
41	1	0	−3.051786	1.389611	−0.293269
42	1	0	−2.237669	3.788905	1.033913
43	1	0	1.890491	2.656001	1.019955
44	1	0	0.311924	3.452966	−1.485369
45	1	0	−1.831520	−0.192904	−1.820206
46	1	0	2.511181	2.020726	−1.930313
47	1	0	4.033305	1.832368	0.715161
48	1	0	6.017862	1.669721	−0.625315
49	1	0	5.128163	1.844268	−2.133817
50	1	0	6.218859	−0.391304	−1.797206
51	1	0	0.803540	−1.562784	0.461198
52	1	0	2.188959	−0.614287	−0.009179
53	1	0	4.834832	−0.319062	0.767166
54	1	0	3.072102	−0.773659	2.381132
55	1	0	1.680118	−1.763537	2.800846
56	1	0	4.983684	−2.140979	2.306358
57	1	0	4.936453	−3.806050	1.675724
58	1	0	4.414861	−0.066134	−3.427437
59	1	0	3.175047	−0.378722	−2.203073
60	1	0	4.254131	−1.676702	−2.723161
61	1	0	5.675420	−2.959209	−1.824052
62	1	0	6.038153	−4.055400	−0.483536
63	1	0	4.391549	−3.940830	−1.087697
64	1	0	−2.299459	4.756041	−1.289836
65	1	0	−3.221512	3.269709	−1.107674
66	1	0	−1.667119	3.235757	−1.936836
67	1	0	−0.339258	5.313902	2.185689
68	1	0	−0.000876	3.633122	2.576240
69	1	0	1.289033	4.670425	1.931669
70	1	0	−7.881045	0.509406	0.235345
71	1	0	−8.608053	−1.570496	−0.892936
72	1	0	−6.983654	−3.389370	−1.384543
73	1	0	−5.518508	0.837099	0.900420
74	1	0	−4.238085	−4.025919	−1.064908
75	1	0	−2.164065	−2.974310	−0.124496
